# Modeling and simulation of amplified spontaneous emission in single-mode-pumped Cr^2+^:ZnSe bulk amplifiers with beam propagation and equivalent input-noise seeding

**DOI:** 10.1007/s00340-025-08433-y

**Published:** 2025-03-01

**Authors:** Johan Nilsson

**Affiliations:** Optoelectronics Research Centre, University of Southampton, Southampton, SO17 1BJ UK

## Abstract

We investigate and use the beam propagation method with equivalent input noise for the simulation of narrow-band amplified spontaneous emission (ASE) and signal amplification in continuous-wave Cr^2+^:ZnSe non-waveguiding “bulk” amplifiers with non-saturating signal and ASE in different configurations with weak reabsorption. Both the incident pump at 1901 nm and the signal at 2410 nm were diffraction-limited gaussian beams. We implemented the equivalent input noise as random realizations of one photon per gridpoint, and showed that this leads to one noise photon per mode. Simulation results of between 100 and 6000 realizations were ensemble-averaged to determine the power spectral density of the ASE in a Monte Carlo approach. We validated the approach by comparing results for single-mode and multimode fiber amplifiers to those obtained with well-established fiber amplifier models. We also calculated the beam quality of the ASE, $\:{M}_{ASE}^{2}$, from its spatial distribution. We found that under some conditions, but not all, $\:{{M}_{ASE}^{2}}^{2}$ can serve as an estimate of an effective number of ASE modes and, together with the ASE PSD, predict the achievable signal gain. It is also possible to evaluate the PSD per unit solid angle due to spontaneous emission from the input noise seeding, and we found agreement down to the single-photon level.

**Supplementary Information:**

The online version contains supplementary material available at 10.1007/s00340-025-08433-y.

## Introduction

Single-mode end-pumping of “bulk” traveling-wave optical amplifiers is attractive since it opens up for tight pump-beam confinement and high gain in relatively long gain media, in which a poor beam-quality would make the pump power prohibitive. Thus, a 17-mm-long Yb: YAG crystal reached a weakly saturated gain of nearly 40 dB at 1030 nm when pumped with 35 W of power at 920 nm from a single-mode Nd-doped fiber laser [1]. In the mid-infrared, Cr^2+^:ZnSe has emerged as a favored material for stimulated emission in a broad wavelength range of around 2–3 μm, peaking at 2.4 μm [2]. Gain as high as 45 dB in the spectral range 2.3–2.6 μm has been reported with pulsed pumping [3]. The pump was an Er-doped fiber laser, few-moded with good beam quality.

**Fig. 1:**
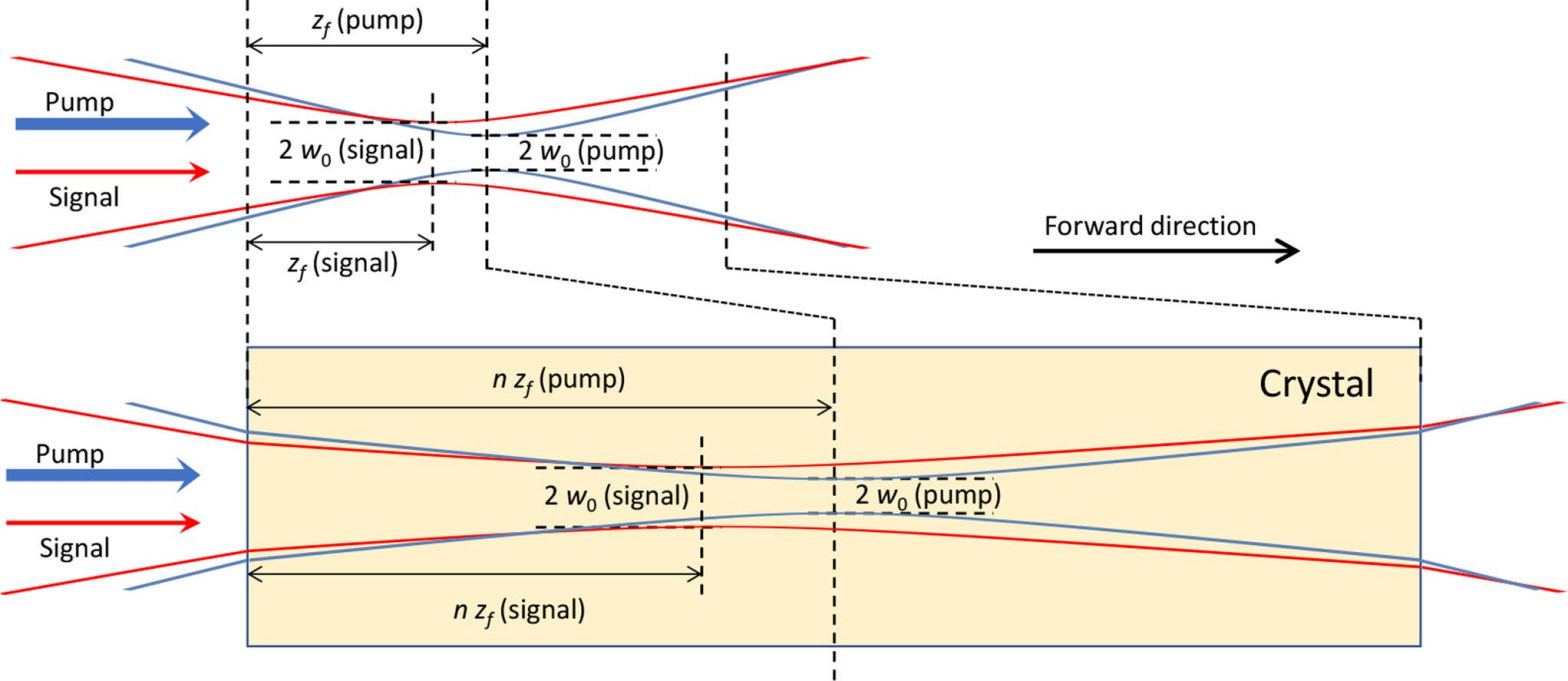
Schematic of the pump and signal beam arrangements, without crystal (i.e., in air; top) and with the Cr^2+^:ZnSe crystal placed in the beams (bottom). The incident beams are collinear, concentric, and diffraction-limited with gaussian profiles and are characterized by the air-values of the beam waist radius *w*_0_ (at *e*^–2^ intensity) and the position *z*_*f*_ of the focal plane relative to the crystal input plane. Disregarding distortions induced by the crystal, the crystal shifts the focal planes to *n z*_*f*_, where *n* is the refractive index. The undistorted pump focal plane is at the crystal midpoint in our simulations. The ASE generated in the crystal is not illustrated

High-gain amplification is accompanied by amplified spontaneous emission (ASE), and the modeling and simulation of ASE in bulk amplifiers with diffraction-limited single-mode pump and signal is the focus of this paper. Figure 1 illustrates the configuration we consider. The drawn beams and their parameters are only examples, except that the undistorted pump focal plane is at the crystal midpoint in our simulations. The ASE generated by the pumped crystal is not shown. ASE is often undesirable (e.g., it adds noise to a signal and can compress the gain and deplete the pump power). A further advantage of the small pump volume of single-mode pumping is that it also reduces the ASE for a given level of signal gain.

Even if undesirable, ASE can also be helpful for assessing the gain spectral shape, bandwidth, and level. For a single mode (including single polarization), the single-sided power spectral density (PSD) of the ASE, *S*_*ASE*_ [W/Hz] is proportional to the inversion factor *n*_*sp*_ (also known as the spontaneous-emission factor), photon energy *hν*, and to (*G*_*lin*_ – 1), where *G*_*lin*_ ≥ 1 is the linear gain. Thus,

1 $$\:{S}_{ASE}\:(\lambda)\:=\:({G}_{lin}(\lambda)\:\--\:1){n}_{sp}\:(\lambda)\:h$$

at some wavelength *λ* in the ASE spectrum. See, e.g. [4–6], (Eq. 58) [7], (Eq. 2.33). We will consider the case when the wavelength is that of a signal to be amplified, but it could be different. The equation applies to waveguiding single-mode amplifiers such as fiber amplifiers as well as to each of the modes of a multimode amplifier. The total PSD of the ASE is given as the sum over all spatial and polarization modes, each with its own value of *G*_*lin*_ and *n*_*sp*_. However, the variations in *n*_*sp*_ may be negligible. For example, in the limit of negligible ground-state absorption (in a four-level system or completely inverted three-level system) and background loss, *n*_*sp*_ = 1. See, e.g. [6], or [7] (Eq. 2.34). Then, *n*_*sp*_ is the same for all modes. The Petermann *K*-factor (also known as the excess spontaneous-emission factor) [5, 6, 8, 9] can also enter into Eq. (1), but we assume that it equals unity.

Alternatively, it is possible to assign an effective number of modes which results in the total PSD when multiplied by *n*_*sp*_ and *G*_*lin*_ – 1 (≈ *G*_*lin*_ at high gain). Although the gain used for such a calculation can be chosen in different ways, we will use the highest gain that the amplifier can attain at a considered wavelength, for a specific set of pump parameters. For *n*_*sp*_, the choice of a representative value is simplified if its modal dependence is small. A complication for non-waveguiding, “bulk,” amplifiers is that the definition of a mode (or modal set) is no longer unique [4]. However, it may still be possible to use the square of the beam propagation factor of the ASE, $\:{M}_{ASE}^{2}$, as a replacement for the effective number of modes [10]. Thus, we hypothesize that for an amplifier that supports multiple modes,

2 $$\begin{aligned}{S}_{ASE}&=\:({G}_{lin}\:\--\:1)\:{n}_{sp}\:{{M}_{ASE}^{2}}^{2}\\&=\:({G}_{lin}\:\--\:1)\:\:{n}_{sp}\:{M}_{ASE,\:x}^{2}\:{M}_{ASE,y}^{2}\end{aligned}$$

where all quantities are evaluated at some wavelength *λ*. The second equality allows for non-circular beams, but our geometry will be circularly symmetric, and we will use the product $\:{M}_{ASE,\:x}^{2}$ $\:{M}_{ASE,y}^{2}$ interchangeably with $\:{{M}_{ASE}^{2}}^{2}$. Another difficulty is that the gain in a bulk amplifier depends on the signal beam alignment, which therefore must be optimal. On the other hand, this makes Eq. (2) potentially more attractive as a means to calculating the highest attainable gain from *S*_*ASE*_ and $\:{M}_{ASE}^{2}$, which may be reasonably simple to measure with standard laboratory equipment. These parameters are therefore interesting to simulate. Note also that the pump configuration is not optimized in our simulations, e.g., in terms of pump waist radius. In other words, our simulations use signal alignments which are first optimized for the different considered pump configurations, though these are in turn generally not optimized.

Simulations of ASE in bulk amplifiers have sometimes assumed that the gain is spatially homogeneous and / or employed ray approximations (ray tracing) [4, 10–14]. However, with diffraction-limited pumping, the high gain and thus the ASE may be restricted to only a few modes. Since diffraction becomes important in this regime, the wave-based beam propagation method (BPM, also known as the Feit-Fleck algorithm) [15–19] has then been used to describe the propagation, e.g., in x-ray / free-electron lasers [17, 19] and other lasers [18]. These are typically mirror-less, thus actually ASE-sources operating with short pulses of high gain. In those simulations, the ASE was seeded by spontaneous emission occurring throughout the pumped gain medium, in bespoke BPM-implementations. The spontaneous emission is a rapidly varying random process, so the ASE varies rapidly in time. Averaging can be used to evaluate, e.g., coherence and stationary and slowly varying parameters, in the pulsed as well as continuous-wave (cw) regime.

As an alternative to seeding the ASE throughout the gain medium, *S*_*ASE*_ can be evaluated by seeding the input of each mode of an amplifier by *n*_*sp*_ input noise photons (equivalent to *n*_*sp*_ photons per second per hertz). See, e.g. [7], (p. 77) [9, 20]. This approach is often used for waveguiding amplifiers and follows directly from Eq. (1). Specifically, *G*_*lin*_ *n*_*sp*_ *hν* = *S*_*ASE*_ + *n*_*sp*_ *hν* when *G*_*lin*_ > 1. When *G*_*lin*_ > > 1, *S*_*ASE*_ ≈ *G*_*lin*_ *n*_*sp*_ *hν*, but more generally, one can just subtract *n*_*sp*_ photons from *G*_*lin*_ *n*_*sp*_ *hν* to arrive at *S*_*ASE*_.

In this paper, we investigate the use of BPM with equivalent input noise of one photon per gridpoint (corresponding to *n*_*sp*_ = 1) and, accordingly, simulate narrow-band ASE (including spontaneous emission) in a cw Cr^2+^:ZnSe amplifier with homogeneous line broadening and low reabsorption. Both the incident pump and the signal are diffraction-limited gaussian beams as illustrated in Fig. 1. We calculate the beam propagation factors and the power spectral density of the generated ASE at the signal wavelength (2410 nm) for different crystal parameters and, as an important objective, assess if Eq. (2) can be used to determine the highest attainable gain, which we also calculate. The ASE and signal are assumed to be weak enough to avoid gain saturation. This was confirmed in most cases. To handle the stochastic nature of the ASE, the results are averaged over between 100 and 6000 realizations in a Monte Carlo method. This approach for simulating the ASE PSD does not require any modes to be defined and can be used with any BPM code, as long as the equivalent input noise is correctly implemented. We validated our approach by comparing BPM simulation results on fiber amplifiers in the single-mode and multimode regime to those of a well-established fiber-amplifier simulation method, as well as to analytic calculations of the spontaneous emission and reach agreement down to the single-photon-level. In addition, we compare different methods for determining $\:{M}_{ASE}^{2}$ from the calculated ASE and find good as well as poor adherence to the hypothesized Eq. (2).

This paper is structured as follows. Section 2 details our simulation approach and parameters. Section 3 validates the approach through comparisons to well-established simulations for optical fiber amplifiers and to analytic calculations of spontaneous emission. We also compare different methods for calculating *M*^2^. Section 4 presents our simulations of Cr^2+^:ZnSe-amplifiers. Section 5 discusses the accuracy of our simulation approach and criteria for the validity of the hypothesized Eq. (2) and summarizes approximate calculations on the amount of saturation by the ASE (“self-saturation”). ASE would only saturate the gain in one of the simulated configurations, and then only for gain of ~ 50 dB or more. In Appendix 1, we show that an equivalent input noise of one photon per gridpoint leads to an equivalent input noise of one photon per mode (or any normalized light distribution).

We do not consider damage limitations and neglect thermo-optic and other refractive-index effects. These can lead to beam aberrations [1, 10, 14] and are often important in Cr^2+^:ZnSe [2], although they can be less significant in the quasi-continuous-wave regime and in gain media with lower thermo-optic coefficient.

To reduce the length of the main text, some less central material is covered in Supplement 1. This includes the numerical grid, the use of a constant inversion factor (*n*_*sp*_ = 1), and the determination of the effective ASE bandwidth which we then use to estimate the ASE self-saturation. It also includes a section on thermally generated radiation. This could be a factor in experiments but is found to be negligible at 2410 nm. Furthermore, we discuss advantages and disadvantages relative to distributed ASE-seeding [9, 17–20] in different regimes.

## Calculation approach

We use BPM as implemented in R P F Power [21] v. 7 to calculate the propagation of a signal wave and an ASE wave, both at a wavelength of 2410 nm, and a pump wave at 1901 nm in the steady-state regime in Cr^2+^:ZnSe crystals. Table 1 lists the parameters we used for the bulk amplifier, as well as parameters used in fiber simulations used for validation, except when otherwise stated. BPM propagates the complex field of a wave in a number of steps, each of which is split into a refractive and a diffractive part (e.g., [15–19]). RP Fiber Power is a commercial software package which uses the paraxial approximation and Fourier transformation for the diffractive part of a BPM propagation step. It does not support seeding of the ASE by spontaneous emission occurring throughout the gain medium. Instead, we used its script language to implement seeding with equivalent input noise with random complex amplitude in the input gridpoints of the BPM simulations. Each gridpoint is seeded with noise of the same complex normal distribution, corresponding to one noise photon (*n*_*sp*_ = 1). Appendix 1 shows that for a waveguide structure, *n*_*sp*_ photons per gridpoint leads to the well-known condition of *n*_*sp*_ equivalent input noise photons per guided mode [5, 7, 9]. This was also confirmed by simulations.

The refractive part of a BPM step includes the amplification and pump absorption by the gain medium. A fraction *n*_2_ of the laser-active ions is in the upper laser level, and the remaining ions are in the lower laser level in our simulations. The local intensities of the propagating waves are used in standard rate equations to calculate the excited fraction, from which the local gain and absorption follow. The numerical grid we use is square with equidistant samples.

BPM is intrinsically monochromatic, so the results are valid for monochromatic pump and signal. Coherence can be important since higher coherence can lead to stronger multipath interference patterns with more pronounced spatial variations of the gain. However, both the pump and signal distributions remain smooth in the gain medium, with only minor interference effects. Furthermore, the signal is assumed to be sufficiently weak to avoid saturation and make spatial hole-burning negligible, so the signal does not influence the propagation of the other waves. Under these conditions, the coherence (or linewidth) is not expected to significantly affect the evolution of the pump and signal.

Also the ASE is assumed to be sufficiently weak to avoid self-saturation and therefore not affect the pump or signal. This means that we can evaluate the ASE PSD (numerically equal to the power in 1 Hz of bandwidth) at only a single wavelength without regard to the rest of the ASE-spectrum. We also disregard backward-propagating ASE with the same motivation and treat all waves as co-propagating in the forward direction. Accordingly, a single BPM run calculates a forward-propagating monochromatic wave related to the ASE PSD for a specific realization of the random noise-seeding. This is then repeated a number of times with different realizations of the random noise-seeding. The spatial intensity-distributions of the different runs are then ensemble-averaged [17–19] to arrive at an uncorrected approximate ASE PSD. The number of runs that is needed depends on the parameters and targeted accuracy. Between 100 and 6000 were found to be sufficient. As a correction, Eqs. (1) & (2) suggest that we should subtract *n*_*sp*_ photons per gridpoint from the ensemble-averaged result. However, a large fraction of the gain medium is unpumped and thus weakly absorbing, e.g., ~ 0.45 dB or ~ 10% in our default bulk crystal. This makes the use of Eqs. (1) & (2) complicated. In fact, because of the absorption in unpumped regions, the power (in 1 Hz) of the equivalent input noise can exceed the uncorrected output when the input grid is large or the gain is low. Therefore, as the correction, we subtract the unabsorbed ~ 0.9 photons (in the default crystal and with *n*_*sp*_ = 1) from the ASE intensity in each point of the output grid. We refer to this as subtraction of residual equivalent input noise (REIN). Given the random nature, this can lead to negative intensities in some gridpoints, but all evaluated quantities of primary interest involve integration of the intensity over several gridpoints, so negative intensity in a few points is acceptable.

After the REIN subtraction, we finally arrive at the spatial distribution of the ASE PSD, from which the beam quality ($\:{M}_{ASE}^{2}$) and PSD (*S*_*ASE*_) can be calculated. Also note that with appropriate scaling of the Fourier transformation, the REIN is the same in real space and in Fourier space (e.g., 0.9 photons per gridpoint), so we subtract the same REIN also in Fourier space (notably for evaluation of the farfield ASE intensity distribution).

For simplicity, we use the approximation *n*_*sp*_ = 1. More precisely, in a homogeneously excited gain medium [4, 6, 7],

3 $$\begin{aligned}{n}_{sp}&=\:{N}_{2}\:{\sigma}_{s}^{e}\:/\:({N}_{2}\:{\sigma}_{s}^{e}\:\--{N}_{1}\:{\sigma}_{s}^{a}\:\--\:\alpha)\hspace{0.17em}\\&=\hspace{0.17em}{n}_{2}\:{\sigma}_{s}^{e}/\:\left[{n}_{2}\:\right({\sigma}_{s}^{e}+\hspace{0.17em}{\sigma}_{s}^{a})\:\--\:{\sigma}_{s}^{a}]\end{aligned}$$

Here, *N*_1_ and *N*_2_ [m^–3^] are the number densities of Cr^2+^-ions in the lower and upper laser level, *σ*^*a*^_*s*_ and *σ*^*e*^_*s*_ are the absorption and emission cross-sections, and *α* [m^–1^] is the background loss. In the second equality, we assume that only two levels are populated and that *α* = 0, which we do throughout this paper. In the presence of reabsorption (*σ*^*a*^_*s*_ > 0), *n*_*sp*_ increases if *n*_2_ and thus the gain decrease. However, because *σ*^*a*^_*s*_ is small compared to *σ*^*e*^_*s*_ at 2410 nm in Cr^2+^:ZnSe, *n*_*sp*_ exceeds 1.1 only for gain below 4.2 dB with our default crystal parameters and uniform excitation. Our gain is generally much higher than that, which justifies the approximation *n*_*sp*_ = 1. See the Supplement 1 for further discussions of *n*_*sp*_ and its use in the BPM input grid.

**Table 1:** Optical and numerical parameters used in simulations

Quantity	Symbol	Value
Pump wavelength		1901 nm
Wavelength for ASE PSD and signal	*λ*	2410 nm
Photon energy of ASE and signal	*hν* or *hν*_*s*_	82.4 zJ
Absorption cross-section at pump wavelength	*σ*_*a*_^*p*^	7.38 × 10^–23^ m^2^
Stimulated-emission cross-section at pump wavelength	*σ*_*e*_^*p*^	3.85 × 10^–23^ m^2^
Absorption cross-section at signal wavelength	*σ*_*a*_^*s*^	0.0873 × 10^–23^ m^2^
Stimulated-emission cross-section at signal wavelength	*σ*_*e*_^*s*^	12.94 × 10^–23^ m^2^
Signal power		Negligible
Inversion factor	*n*_*sp*_	1
**Fiber parameters**		
Pump power		200 kW (essentially infinite)
Fiber length		50 mm
Cr^2+^-concentration		3.66 × 10^24^ m^–3^
Refractive index of cladding		2.45
Core numerical aperture		0.0313
Core radius, SMF		23.19 μm
Core radius, MMF		85.77 μm
**Bulk crystal parameters**		
Crystal length		17 mm
Refractive index	*n*	2.45
Cr^2+^-concentration		7.03 × 10^24^ m^–3^ (Case A, C, D) 14.05 × 10^24^ m^–3^ (Case B)
**Grid parameters**		
Transverse spacing of gridpoints	Δ*x* = Δ*y*	16 μm (Case A & B) 8 μm (Case C & D and fiber simulations)
Size of transverse window		512 μm (Case A & B) 2048 μm (Case C & D and fiber simulations)
Step length		50 μm (fiber simulations) 85 μm (Case A) 42.5 μm (Case B) 100 μm (Case C & D)

The propagated ASE field and thus its PSD *S*_*ASE*_ that we calculate include spontaneous emission, *S*_*sp*_. This can be measured and is also readily evaluated from simulated and / or measured quantities such as the absorbed pump power and the number of excited ions, when reabsorption can be neglected. In cases and regions where the emission has experienced little amplification, *S*_*sp*_ may even dominate over the contribution from stimulated emission to *S*_*ASE*_. This is true even though only the fraction of the spontaneous emission emitted at angles supported by the numerical grid contributes to *S*_*ASE*_, as calculated with BPM. This fraction depends on the transverse grid spacing and was of the order of 0.1%.

Whereas *S*_*ASE*_ is just a simple sum over all points in the output numerical grid, the calculation of $\:{M}_{ASE}^{2}$ is more intricate. We found the details to be critical and considered three different calculation methods, based on the ensemble-averaged ASE intensity distributions at the exit plane of the gain medium and in the farfield.

To calculate the farfield distribution, we implemented a lens as a parabolic phase function at the crystal exit plane for the purpose of minimizing the divergence of the averaged beam, so that the exit plane coincides with the waist of the beam after the lens. We Fourier-transformed the lensed field (i.e., the complex amplitude) for each realization. The overall farfield intensity-distribution was calculated as the ensemble-average of the intensity-distributions of all the realizations (with REIN subtraction). This process was performed for a range of focal powers, and the focal power yielding the smallest divergence was ultimately selected.

One method we used to calculate the exit-plane beamwidth, divergence, and subsequently $\:{M}_{ASE}^{2}$, was a second-moment (“*D*4*σ*”) approach similar to ISO 11,146 [22]. A second method corresponds to a slit scanned across the beam in orthogonal directions, whereby the beamwidth is determined from the positions on different sides of the peak at which the value reaches some fraction of the peak value. We used full-width at half-maximum (FWHM) values and divided those by (2 ln 2)1/2 ≈ 1.177. In case of a diffraction-limited gaussian beam, this converts the FWHM-values to the 2nd -moment values of the radius *w*_0_ and divergence half-angle *θ*_0_. Other levels are also possible, for example, a fractional intensity level of 1/*e*^2^ is often used. Although the level is important, we did not consider it carefully, but found that a lower level led to better agreement in Eq. (2) in one investigated case.

Our beams are expected to be circularly symmetric and in a third method, “circle-scan”, we evaluated the quantity $\:{I}_{c}\left(r\right)=\frac{1}{2\pi\:}{\int\:}_{0}^{2\pi\:}I\left(r\text{cos}\theta\:-{x}_{0},\:r\text{sin}\theta\:-{y}_{0}\right)d\theta\:$ in polar coordinates (*r*, *θ*). Here, *I* (*x*, *y*) is the ASE PSD distribution in the exit plane and (*x*_0_, *y*_0_) is the beam center-of-mass. The units of *I* and *I*_*c*_ are W Hz^–1^ m^–2^. The beam radius was then taken to be the value of *r* where *I*_*c*_ (*r*) reaches 1/*e*^2^ of its peak value. The divergence was similarly evaluated from the farfield.

With these choices of fractional intensity levels, circle-scanning led to $\:{M}_{ASE}^{2}$-values that adhered better to Eq. (2) than slit-scanning did. The ISO-approach proved the worst in this respect.

We ignore Fresnel reflections. These can be significant, e.g., 17.7% at a refractive index of 2.45, but can be reduced by an anti-reflection coating.

Our simulations neglect thermo-optic (thermal-guiding) and polarization effects. As it comes to the PSD of the ASE, our calculations are for a single polarization. The pump and signal polarizations are irrelevant for Cr^2+^:ZnSe and other isotropic gain media, and the total ASE is twice that in a single polarization. Since there are no nonlinear effects for the ASE and signal (including saturation), their propagation is linear in our simulations.

Thermo-optic effects (extending to stress-optic effects and bulging of end-facets) and resulting beam aberrations are often important in Cr^2+^:ZnSe [2], although they can be less significant in the quasi-continuous-wave regime and in gain media with lower thermo-optic coefficient. We expect that the inclusion of such effects, as well as ASE self-saturation and other nonlinearities, to be challenging and computationally intensive. The nonlocal nature and delayed response of thermo-optic effects contribute to the difficulty. Iterative approaches may well be required, and these can have problems with convergence. Any notional solution may even be unstable. Having said that, input-end noise seeding is fundamentally compatible with BPM solvers that include nonlinearities such as thermo-optic effects, e.g., if this capability is added to an updated version of RP Fiber Power (stimulated Raman scattering and the nonlinear Kerr effect are already available in RP Fiber Power).

BPM with thermo-optic effects is treated in [23]. Reference [19] also discusses some related issues. Both references describe temporally resolved simulations, which although computationally demanding can overcome problems related to the simulation of thermal diffusion and convergence with bidirectional saturating waves including ASE. Ultimately, the choice of method depends on what nonlinear effects are considered. Input-end noise seeding may be compatible with all nonlinear effects and simulation methods, but even if so, it may not be best.

We also point out that although the implementation of the noise seeding was in itself relatively straightforward, the overall programming effort was considerable. There were some 2200 lines of code in the RP Fiber Power script language, with large fractions devoted to loops and post-processing (to calculate *M*^2^-values of the signal as one example), writing output files, plotting, etc. This was a modification and extension of a script for the simulation of a bulk amplifier kindly provided by Dr. Rüdiger Paschotta. Mathematica was used for plotting and further postprocessing of the output from RP Fiber Power, e.g., for ensemble-averaging, calculating *M*^2^-values of the ASE in different ways, and identifying optimal signal parameters. This comprised ~ 1500 lines of code.

## Validation through simulations of fiber amplifiers

We simulated fiber structures with a well-defined number of guided modes and well-defined gain confined to the core with BPM and compared the results to those obtained with conventional equations for the evolution of modal power in the incoherent regime, which we refer to as “modal power evolution”, MPE. See, e.g., [7, 20, 24]. The MPE-equations are well proven for the simulation of gain and ASE in fiber amplifiers and can therefore be used for validation. We used RP Fiber Power also for the MPE simulations. In contrast to our BPM simulations, the MPE-implementation seeds the ASE with spontaneous emission distributed along the fiber. The MPE-calculations used analytic *LP*-modes. The BPM-calculations used a configuration like that in Fig. 1, with diffraction-limited gaussian input beams. These were now focused on the fiber input face (*z*_*f*_ = 0), with beam waist radii *w*_0_ obtained with Marcuse’s formula for the mode field radius of the fundamental mode [25].

We simulated a single-mode fiber and a multimode fiber. Both fibers were 50-mm long and had a cladding refractive-index of 2.45. The cores had a numerical aperture (NA) of 0.031 and were Cr^2+^-doped with a concentration of 3.66 × 10^24^ m^–3^. The cross-sections for stimulated emission and absorption, as well as the pump wavelength (1901 nm) and signal / ASE wavelength (2410 nm), were set to the values used for the Cr^2+^:ZnSe bulk crystal (Table 1). These parameters are not realistic for a fiber but are fine for validation and largely agree with those of the bulk crystal. The pump power was 200 kW in the fiber simulations. This choice is also unrealistic but ensures that *n*_2_ is clamped at the pump transparency level of *σ*_*a*_^*p*^ / (*σ*_*a*_^*p*^ + *σ*_*e*_^*p*^) = 0.657. At this excitation level, the gain becomes 1.35 dB/mm if all of the signal propagates within the gain medium (unity overlap). Furthermore, from Eq. (3), *n*_*sp*_ = 1.0035, which is only 0.015 dB higher than *n*_*sp*_ = 1 used in the BPM simulations.

The BPM simulations further used a longitudinal step length of 50 μm and 256 × 256 transverse points with a spacing of Δ*x* = Δ*y* = 8 μm (window size 2048 μm). The grid thus supports propagation angles up to *θ*_*g*_ = *λ* / (2 *n* Δ*x*) = 61.5 mrad (in the *x*-direction) in the paraxial approximation at an in-fiber wavelength of 2410 nm / 2.45 = 984 nm. Note also that 256 × 256 = 65,536 transverse points, so with seeding of one noise photon per point in the input grid, the total seeding is 48.2 dB higher than the single-photon seeding of a single mode.

In these simulations, only the pump (which is forward-propagating) affects the excitation level, and there is no gain-saturation from backward-propagating light. Thus, the forward-propagating waves at a longitudinal position *z* are independent of backward-propagating waves at that position, and independent of what happens at locations further forward. It follows that the output parameters (e.g., power) from a fiber of a specific length *L* is equal to the value of those parameters at *z* = *L* in a longer fiber. We took advantage of this in the MPE-simulations but not in the BPM simulations, to save on scripting efforts.

### Single-mode fiber amplifier

**Fig. 2:**
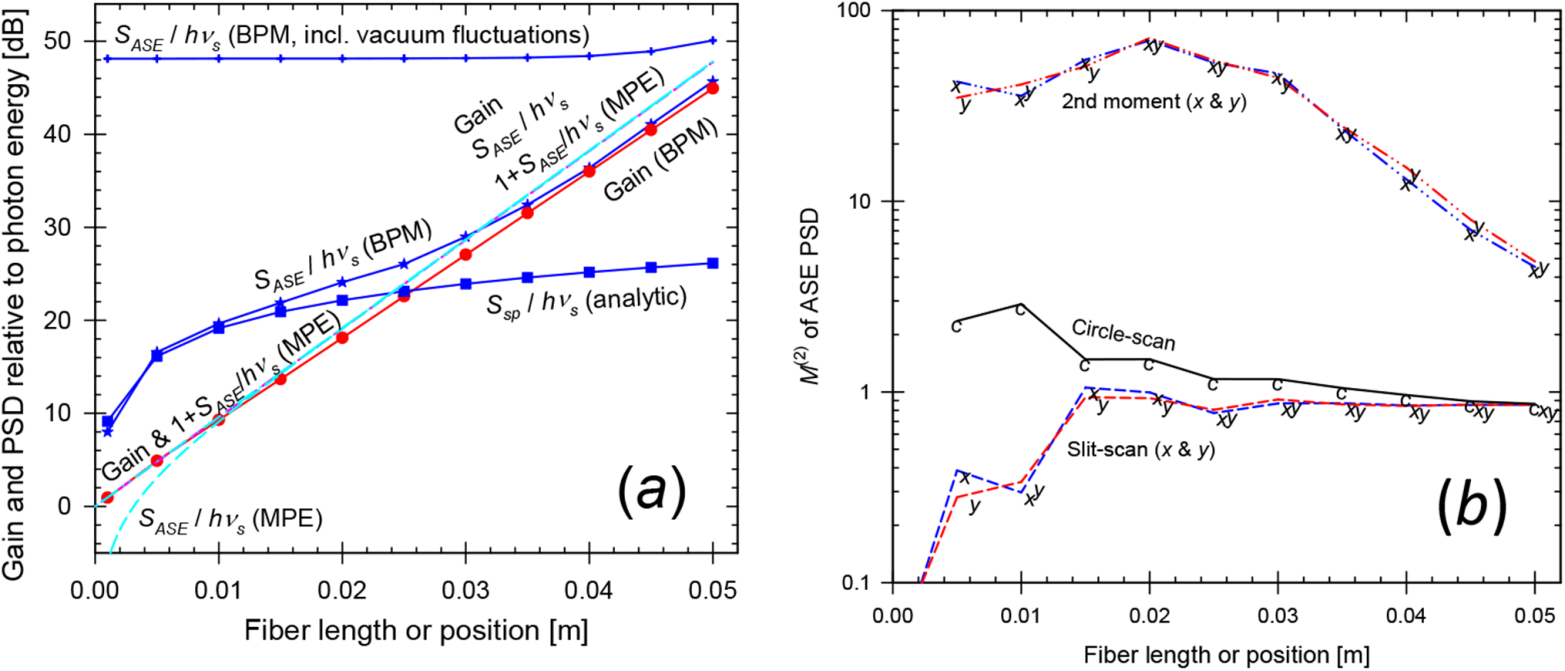
Single-mode fiber. (**a**) The gain, ASE level *S*_*ASE*_ / *hν* (before and after subtraction of residual equivalent input noise) and *S*_*ASE*_ / *hν* + 1 simulated with BPM and MPE as indicated. For the MPE-simulations, the curves for *S*_*ASE*_ / *hν* + 1 and the gain are indistinguishable. The analytically calculated spontaneous-emission level *S*_*sp*_ is also shown. (**b**) $\:{M}_{ASE}^{2}$ from BPM-simulations, evaluated as a 2nd -moment (*x* and *y*), with slit-scanning (*x* and *y*), as well as with circle-scanning

The single-mode fiber had a 46.38-µm diameter core with *V* = 2.40 at the pump wavelength. For the BPM-calculations, the waist radii *w*_0_ were 25.52 μm and 30.94 μm for pump and signal. For the MPE-calculations, both the pump and signal were launched as *LP*_01_-modes. Figure 2 shows how the small-signal gain and ASE evolves along the fiber, with the two methods. The ASE is plotted in terms of *S*_*ASE*_ / *hν*, i.e., the ASE PSD at the signal wavelength in one polarization, relative to the signal photon energy. For the MPE calculations, this practically coincides with the signal gain for gain over ~ 13 dB. At lower gain, *S*_*ASE*_ / *hν* + 1 still coincides with the gain, thus following Eq. (1).

The gain calculated with BPM is similar to that calculated with the MPE equations. The 6% reduction in logarithmic gain (the dB gain) in Fig. 2 may be caused by the sampling, i.e., a lower overlap in the BPM grid. With each gridpoint representing an area of Δ*x* Δ*y* = 64 µm^2^, the core area corresponds to 26.40 gridpoints, but the sampling was such that only 25 gridpoints were within the core. We also see that the ASE-level in the BPM calculations agrees with that of the MPE-calculations for gain above around 24 dB. Furthermore, for BPM-calculations, the ASE and gain levels are within 2 dB of each other, when the gain exceeds ~ 25 dB of gain (*G*_*lin*_ ≈ 320). Thus, *S*_*ASE*_ is within 2 dB of ~ 320 photons at *G*_*lin*_ = 320. This is 23 dB lower than the 65,536 of residual photons in the unpumped output (i.e., the REIN), which we subtract from the ASE intensity. This indicates that our subtraction of the residual equivalent input noise is accurate at that point. Note that in our fiber simulations, the number of photons in the REIN is practically the same as the number of input noise photons, since the average doping of the simulated structure and thus the absorption of the input noise photons across the whole grid is negligible (the doped core makes up only a fraction of 4.03 × 10^–4^ of the simulated volume).

For lower gain, unguided spontaneous emission (which may experience a small amount of amplification insofar as it overlaps with the core) increases relative to the ASE. The spontaneous emission couples primarily to the large number of unguided modes, including those supported by the BPM grid. Thus, the spontaneous emission contributes much more to *S*_*ASE*_ as calculated with BPM than to that calculated with MPE, which only includes the spontaneous emission captured by the guided mode. Therefore, *S*_*ASE*_ will differ. The PSD of the spontaneous emission, *S*_*sp*_ (without any amplification) is also shown in Fig. 2. We calculated this analytically, as the PSD generated per excited ion and unit solid angle in one polarization, *hν n*^2^ *σ*_*e*_^*s*^ / *λ*^2^ (from [26]) multiplied by the solid angle supported by the BPM grid (= 4 × 0.0615^2^ sr = 0.0151 sr in the paraxial approximation) and the number of excited ions. Within 1 dB, *S*_*sp*_ agrees with *S*_*ASE*_ at low gain, and we conclude that *S*_*ASE*_ as calculated with BPM is dominated by spontaneous emission at low gain. Figure 2 shows that the calculated PSD (*S*_*ASE*_) remains correct even when it becomes nearly 40 dB lower than the uncorrected PSD (before REIN subtraction), in regimes where the PSD is dominated by spontaneous emission and the amplification of the spontaneous emission is low. This further confirms the accuracy of the REIN subtraction.

The beam propagation factors $\:{M}_{ASE}^{2}$ are plotted in Fig. 2 (b), as evaluated from the BPM-simulations in the three different ways. For an analytic *LP*_01_-mode, *M*^2^ is close to unity, e.g., < 1.1. The 2nd -moment calculation yields $\:{M}_{ASE}^{2}$-values which are much larger. We attribute this discrepancy to low levels of background light at large distances from the core, which is known to increase the calculated *M*^2^-value (e.g., [27]). In further calculations (not shown), the second-moment calculation came close to expected values of $\:{M}_{ASE}^{2}$ for gain > 70 dB, when the background is sufficiently low relative to the light guided by the core. By contrast, the slit-scan and circle-scan determinations of $\:{M}_{ASE}^{2}$ seem reasonably accurate for gains of ~ 12 dB or more, which is reached at around 15 mm of propagation. We also found that the transverse profile of the signal (launched with a gaussian profile), and thus its *M*^2^-value, stabilized after around 20 mm of propagation.

**Fig. 3:**
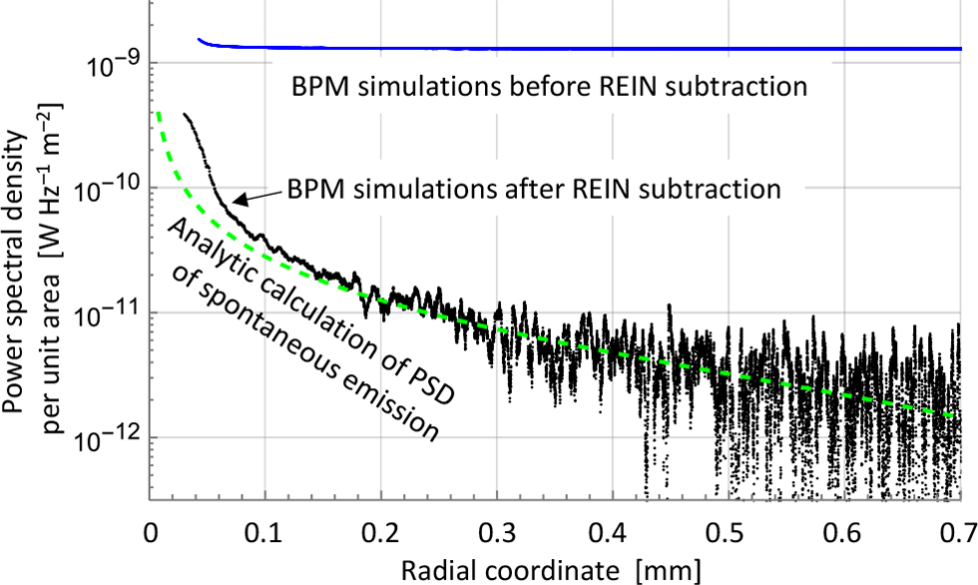
Single-mode fiber, 15 mm. Power spectral density per unit area according to analytically calculated spontaneous emission as well as BPM simulations before and after REIN subtraction

To further test the validity of the BPM-calculations and the subtraction of the REIN, we compared the spatial distribution of the calculated PSD [W Hz^–1^ m^–2^] to analytic calculations of the spontaneous emission from 15 mm of fiber. For this, *S*_*ASE*_ was averaged over 4000 runs. Figure 2 (a) suggests that spontaneous emission may dominate the calculated *S*_*ASE*_ at this length. Furthermore, at this length, the simplifying assumptions of negligible amplification and boundary effects that these analytic calculations rely on are fulfilled in a reasonably large part of the output field. Note also that there is no reabsorption in the undoped cladding. The analytic evaluation only included the emission in the angular range supported by the BPM grid and a single polarization (like the BPM simulations). Figure 3 shows *I*_*c*_ (*r*) (defined previously) before and after REIN subtraction, as well as the analytic evaluation. For small radial coordinates, the BPM calculation is dominated by light that has traveled in the core or through the core at a small angle over a relatively long length (e.g., up to 4.6 mm at 10 mrad), thus experiencing significant amplification. This results in stronger ASE which is not captured by the analytic calculation. Light at larger radii has traveled at larger angles and is thus less affected by amplification. Radial positions > 0.15 mm show good agreement, and we conclude that following REIN subtraction, the BPM PSD distribution agrees well with the spontaneous emission distribution in regions with little ASE.

Whereas the REIN does not contribute to the actual ASE and is therefore subtracted, the spontaneous emission does contribute, e.g., in the form of a spatially wide background (56% of the power is outside the core in the BPM simulations of Fig. 3). This background can affect $\:{M}_{ASE}^{2}$, and the 2nd -moment value is particularly sensitive. We note that the spatial distribution of the spontaneous emission can be affected also by factors such as cladding diameter and coating loss, which we did not consider and are often not included in simulations.

From these calculations on single-mode fibers, we conclude that our approach for the simulation of signal gain and ASE is fundamentally correct and accurate at sufficiently high gain. The ASE PSD as calculated with our BPM approach is within 2 dB of that obtained with the standard MPE-model for fiber amplifiers for gain > 25 dB. Furthermore, the relation between the gain and ASE PSD agrees with Eq. (1) for gain > 24 dB. Similarly, the beam propagation factor of the ASE agrees with the expected $\:{M}_{ASE}^{2}$ ≈ 1 when the gain is sufficiently high. This was for gains above ~ 12 dB in case of circle-scanning and slit-scanning but gain as high as 70 dB was required for the 2nd -moment method. We attribute the deviations at lower gain to background light in the form of unguided spontaneous emission. At low gain, there is good agreement between *S*_*ASE*_ and the PSD calculated analytically for spontaneous emission, both regarding their total level and their distribution. Our results also highlight the significance of accurately subtracting the residual unpumped background, which we succeeded with.

### Multimode fiber amplifier

**Fig. 4:**
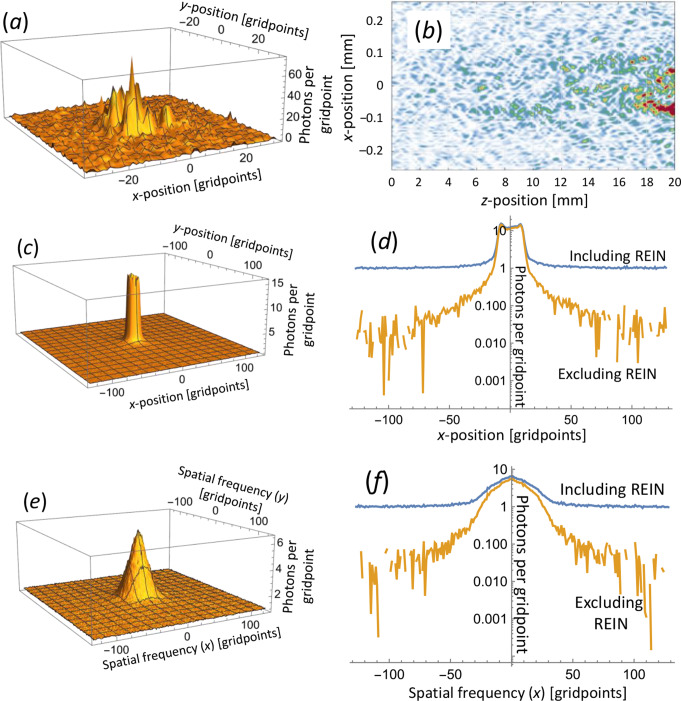
Multimode fiber, 20 mm. Examples of ASE PSD distribution in spatial and angular (spatial frequency) domain calculated with BPM. (**a**) Output intensity of a single realization with random input noise including REIN. (**b**) Intensity of a single realization along the fiber cut in the plane *y* = 0. (**c**) Output intensity (including REIN) ensemble-averaged over 1000 realizations. (**d**) Cut through center of (*c*) at *y* = 0 before and after REIN subtraction. (**e**) Farfield intensity distribution (i.e., in spatial-frequency domain) ensemble-averaged over 1000 realizations. The plot includes the REIN of one photon per gridpoint. The divergence measured as the circle-scanned radius has been minimized with a lens. (**f**) Cut through center of (**e**) before and after REIN subtraction

**Fig. 5:**
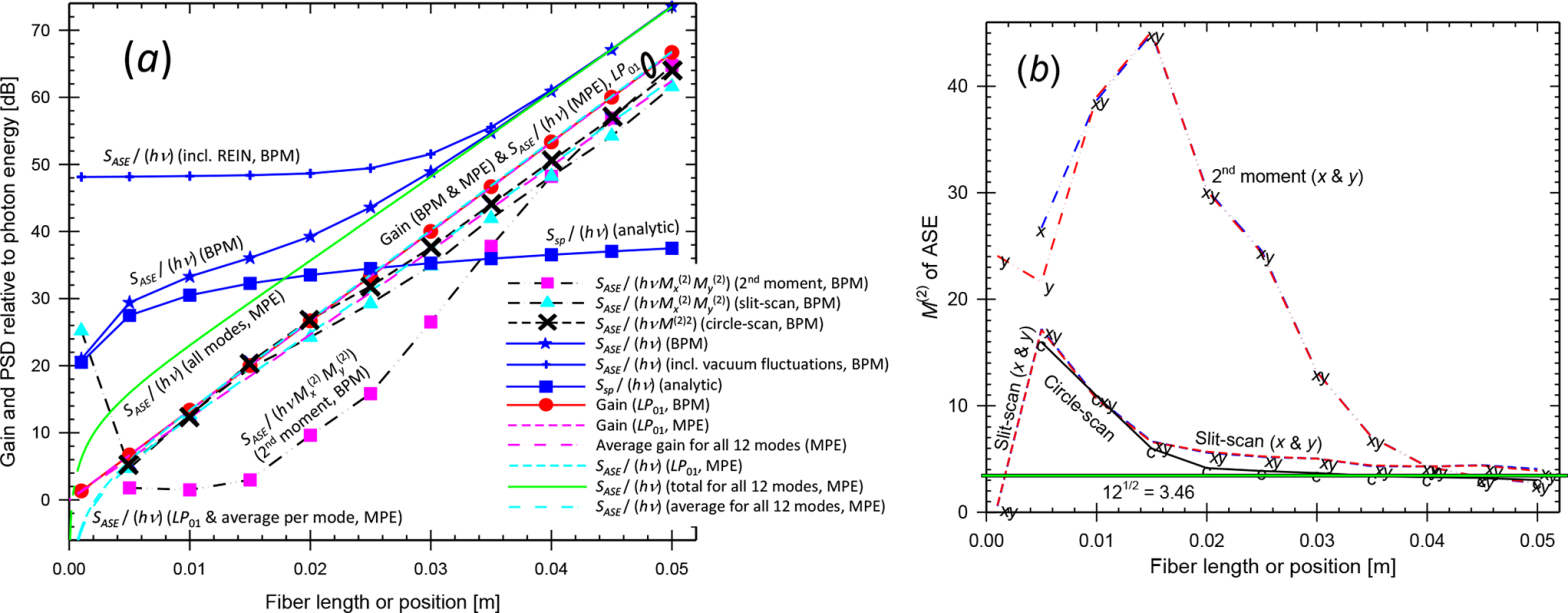
Multimode fiber. (**a**) Gain, ASE level *S*_*ASE*_ / *hν* before and after subtraction of residual equivalent input noise and *S*_*ASE*_ / (*hν* $\:{{M}_{ASE}^{2}}^{2}$) for BPM and MPE-simulations. Quantities are shown, selectively, for *LP*_01_, all modes, and average per mode. *S*_*ASE*_ / (*hν* $\:{{M}_{ASE}^{2}}^{2}$) (from BPM simulations) is depicted for circle-scan determinations of $\:{M}_{ASE}^{2}$ as well as for slit-scan and 2nd -moment determinations. Some curves appear indistinguishable. The analytically calculated spontaneous-emission level *S*_*sp*_ is also shown. (**b**) $\:{M}_{ASE}^{2}$ from BPM-simulations, evaluated as a 2nd -moment (*x* and *y*), with slit-scanning (*x* and *y*), and with circle-scanning. The value of 3.46, approximately corresponding to 12 modes, is indicated

Next, we consider a multimode fiber amplifier. The physical and numerical parameters are the same as for the single-mode fiber, except for the larger core diameter of 171.5 μm. The core supports 12 LP-modes at the signal wavelength (*V* = 7.00). For the BPM-calculations, the waist radii *w*_0_ of the incident gaussian beams were 61 μm (pump) and 63.25 μm (signal), which match the radii of the fundamental modes. These are well confined to the core for *V* = 7.00. The high pump power (200 kW) means the intensity suffices to excite the Cr^2+^-ions to the pump transparency level even at the edge of the core. There are 357 gridpoints within the core. Figure 4 illustrates results of BPM simulations for 20 mm of propagation. Figure 5 shows the calculated gain and *S*_*ASE*_ (BPM & MPE), $\:{M}_{ASE}^{2}$ (BPM), and *S*_*sp*_ (analytic). When all modes are excited by the same signal power, the overall gain for 50 mm calculated with MPE becomes 62.5 dB, which can be viewed as the average modal gain. The *LP*_01_-gain (MPE) reaches 66.6 dB, which closely approximates the potential gain of 67.4 dB with unity overlap. The BPM simulations yield a gain in close agreement (66.7 dB). The second-moment *M*^2^-value for the signal (i.e., not ASE) becomes 1.06, consistent with the supposition of fundamental-mode propagation in the BPM simulations.

Along the length of the fiber (Fig. 5), the MPE-calculations lead to an ASE PSD (relative to the photon energy) in *LP*_01_ which agrees closely with the *LP*_01_-gain once this exceeds ~ 13 dB (distances > 10 mm). As with the single-mode fiber, the agreement extends also to lower levels of gain, if an extra photon is added to the ASE in *LP*_01_. Furthermore, the total ASE PSD becomes 12 times the average gain for values above ~ 13 dB, i.e., the MPE-calculations lead to an average ASE per mode that agrees with the average gain.

When it comes to the BPM-calculations, the resulting ASE PSD agrees with that of the MPE-calculations for all modes within 3 dB for average gain above 27 dB and within 1 dB above 36 dB. For higher gain the curves become indistinguishable. Thus, our method for calculating the ASE PSD with BPM is valid for high gain. For low gain, unguided spontaneous emission appears to contribute significantly to *S*_*ASE*_. As demonstrated for the single-mode fiber, *S*_*ASE*_ may still be correctly calculated but is no longer dominated by core-guided ASE. The MPE-calculations do not include *S*_*sp*_, leading to a lower value of *S*_*ASE*_.

We next consider whether the quantity $\:{{M}_{ASE}^{2}}^{2}$ (or $\:{M}_{ASE,\:x}^{2}$ $\:{M}_{ASE,y}^{2}$) can be used for the effective number of modes, as proposed in Eq. (2). The 12 modes in our fiber would correspond to $\:{M}_{ASE}^{2}$ (and $\:{M}_{ASE,\:x}^{2}$ and $\:{M}_{ASE,y}^{2}$) of approximately 12^1/2^ ≈ 3.46. However, since the lower-order modes with lower *M*^2^-values tend to be more confined to the core and thus reach higher gain and output power, the overall $\:{M}_{ASE}^{2}$ is weighted towards the modes with lower *M*^2^-values. Figure 5 (b) shows values of $\:{M}_{ASE}^{2}$ from the BPM-simulations. The second-moment values of $\:{M}_{ASE}^{2}$ become 2.73 and 2.79 for the two orthogonal directions at 50 mm. This suggests $\:{M}_{ASE,\:x}^{2}$ $\:{M}_{ASE,y}^{2}$ = 7.63 effective modes. The *M*^2^-values are larger for shorter propagation distances (with lower gain), e.g., 45 at 15 mm. This high value is attributed to power in a spatially wide background and suggests that the second-moment calculation should not be used in Eq. (2).

With a slit-scan determination (FWHM), the two orthogonal $\:{M}_{ASE}^{2}$-values become 4.05 and 3.87 at 50 mm. Their product becomes 15.7, so the $\:{M}_{ASE}^{2}$-values determined this way seem to over-estimate the number of modes, even though the background errors should be relatively unimportant, given the high gain at 50 mm for light in the core. Circle-scanning (threshold 1/*e*^2^) yields $\:{M}_{ASE}^{2}$ = 3.02, suggesting 9.13 effective modes. The value remains below 12^1/2^ for distances > 33 mm (44 dB of *LP*_01_-gain). The slit-scanned and circle-scanned values of$\:{M}_{ASE}^{2}$ increase slowly for shorter lengths, down to 15–20 mm. This may be a result of reduced spatial gain-peaking. For even shorter lengths, they increase rapidly, which suggests a significant fraction of the light is outside the core. Still, they remain much smaller than the 2nd -moment values.

Figure 5 (a) also shows the quantity *S*_*ASE*_ / $\:{{M}_{ASE}^{2}}^{2}$, which we compare to the highest achievable single-mode gain (i.e., the gain of *LP*_01_ in this case), according to Eq. (2). If we use Eq. (2) to calculate $\:{{M}_{ASE}^{2}}^{2}$ from *S*_*ASE*_ and *G*_*lin*_, we get $\:{{M}_{ASE}^{2}}^{2}$ = 4.91 = 2.22^2^ at 50 mm. All the $\:{M}_{ASE}^{2}$-values determined from the ensemble-averaged BPM intensity distributions were larger than 2.22, but are reasonably close for the 2nd -moment and circle-scan evaluations. However, for lengths < 40 mm, the 2nd -moment $\:{M}_{ASE}^{2}$-values become large, and *S*_*ASE*_ / $\:{{M}_{ASE}^{2}}^{2}$ underestimates the *LP*_01_-gain. By contrast, the circle-scan determination leads to values of *S*_*ASE*_ / $\:{{M}_{ASE}^{2}}^{2}$ that remain within 3 dB of the *LP*_01_-gain for all lengths down to 5 mm. The slit-scan calculations adhered somewhat worse to Eq. (2), especially at high gain. See Fig. 5 (a).

**Fig. 6:**
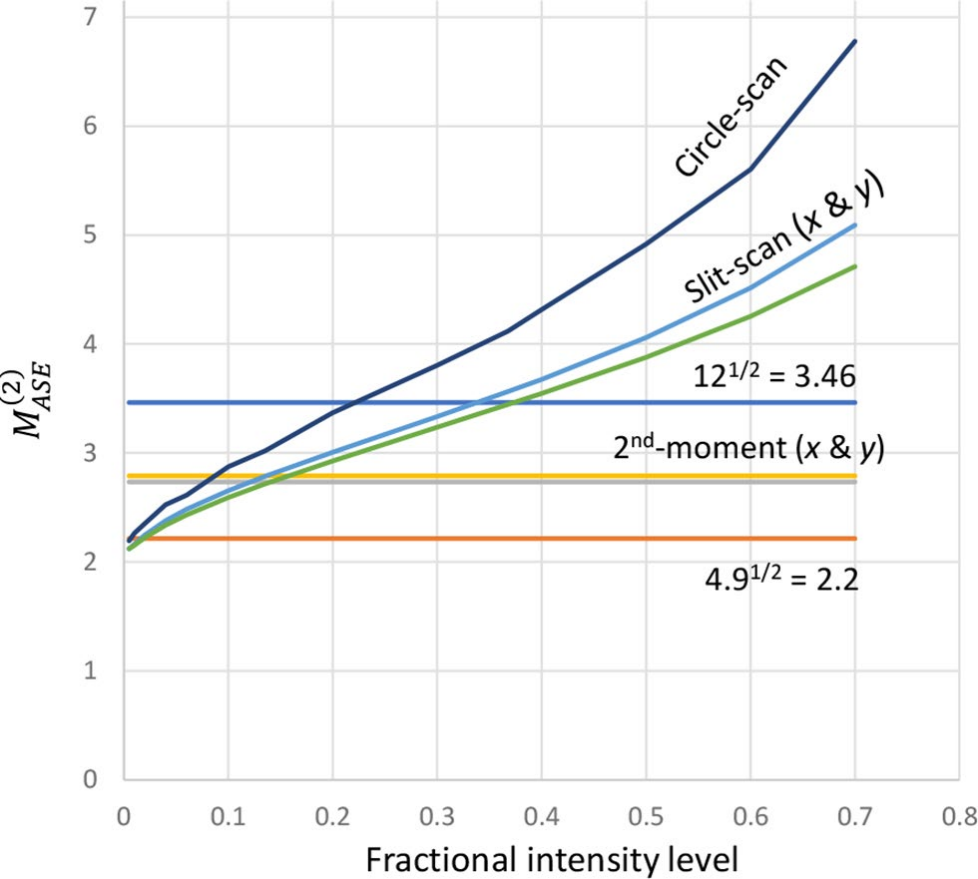
Multimode fiber, 50 mm. $\:{M}_{ASE}^{2}$ calculated with exit-plane beam radii *w*_0,*ASE*_ and farfield divergence half-angles *θ*_0,*ASE*_ determined from slit-scans and circle-scans at different fractional intensity levels and from their 2nd moments (for which the intensity-level does not apply)

Here as well as in other simulations, we generally found the circle-scan evaluation of $\:{M}_{ASE}^{2}$ to conform best to Eq. (2), but this may be due to the choice of the fractional intensity level *I*_*ref*_ used for determining the beam diameter rather than the method itself. We briefly investigated this, by calculating slit-scan and circle-scan $\:{M}_{ASE}^{2}$-values for the 50-mm fiber determined with different values of *I*_*ref*_. The directly determined ASE diameters 2 *w*_0,*ASE*_ and full-angle divergences 2 *θ*_0,*ASE*_ were divided by (–2 ln *I*_*ref*_)^1/2^ before calculating $\:{M}_{ASE}^{2}$ (= *π w*_0,*ASE*_ *θ*_0,*ASE*_ / *λ*). This leads to *M*^2^ = 1 for a diffraction-limited gaussian beam. Figure 6 shows the result, together with the 2nd -moment calculation as well as the values of $\:{M}_{ASE}^{2}$ that correspond to 12 modes and 4.9 modes (taking the number of modes as $\:{{M}_{ASE}^{2}}^{2}$). We see that for these parameters, the reason the circle-scan leads to a lower value of $\:{M}_{ASE}^{2}$ is that it is determined at a lower fractional intensity level, 1/*e*^2^ = 0.135 vs. 0.5 for the slit-scanning. Different fractional intensity levels may fit better with Eq. (2) in general, but we did not investigate this further. These $\:{M}_{ASE}^{2}$-values are all from BPM simulations. We did not calculate $\:{M}_{ASE}^{2}$ for the MPE-simulations.

Overall, we conclude that our BPM-approach for calculating the guided ASE PSD is correct also in these simulations of a multimode fiber for gain > 27 dB, verified through comparisons to MPE simulations. Also unguided spontaneous emission (including weakly amplified spontaneous emission) appears to be correctly calculated. Furthermore, Eq. (2) is correct to within 3 dB, when $\:{M}_{ASE}^{2}$ is determined with circle-scanning with fractional intensity level of 1/*e*^2^. The accuracy is better at higher gain, whereas deviations can become large at low gain. We reiterate that second-moment-calculations of $\:{M}_{ASE}^{2}$ generally led to large discrepancies in Eq. (2). This may be a result of the spontaneous-emission background. For circle-scanning and slit-scanning, the choice of fractional intensity level used for the determination of beam radius and divergence is important but was not studied in depth.

## Simulations of Cr2+:ZnSe amplifiers

We now turn to non-waveguiding “bulk” Cr^2+^:ZnSe amplifiers in the arrangement depicted in Fig. 1. We simulate these in the small-signal regime using BPM in four different cases. Each case involves the pump being focused at the center of the gain medium (crystal), with variations in waist radius and/or dopant concentration, and ignoring any distortions induced by the crystal. To test the validity of Eq. (2), we need to determine the highest achievable gain for any signal beam launched through the crystal under the different conditions. Therefore, optimization of the signal launch is necessary, whereas optimization of the pumping or crystal length is not required for testing Eq. (2). We limit our optimization efforts to pump and signal beams which would be collinear and concentric diffraction-limited gaussian beams, in the absence of gain- and absorption-induced aberrations in the crystal. Although the crystal does aberrate the beams, we anticipate that the gain achieved with these restrictions will be close to what could be attained with unrestricted signal optimization (e.g., allowing for non-gaussian beams).

With these restrictions, we used BPM to simulate and optimize the signal gain with respect to the undistorted air-values of the signal waist radius *w*_0,*s*_ and focal plane position *z*_*f*,*s*_ for different pump powers. Subsequently, we evaluated Eq. (2) with the optimized gain, along with the ASE PSD and *M*^2^-values determined through BPM simulations of the ASE (with ensemble-averaging and REIN subtraction). Note that in the small-signal regime we consider, the ASE is independent of the presence of the signal, and vice versa. The amplifiers consisted of 17 mm of Cr^2+^:ZnSe. Given a refractive index *n* of 2.45, the undistorted air-value of the pump focal position *z*_*f*,*p*_ becomes 8.5 / 2.45 = 3.47 mm for mid-point focusing. Except for Case B below, the Cr^2+^-concentration was 7.03 × 10^24^ m^–3^. With our pump and signal wavelengths, this leads to a gain of 2.59 dB/mm with unity overlap and thus 44.0 dB in 17 mm in the limit of infinite pump power.

**Fig. 7:**
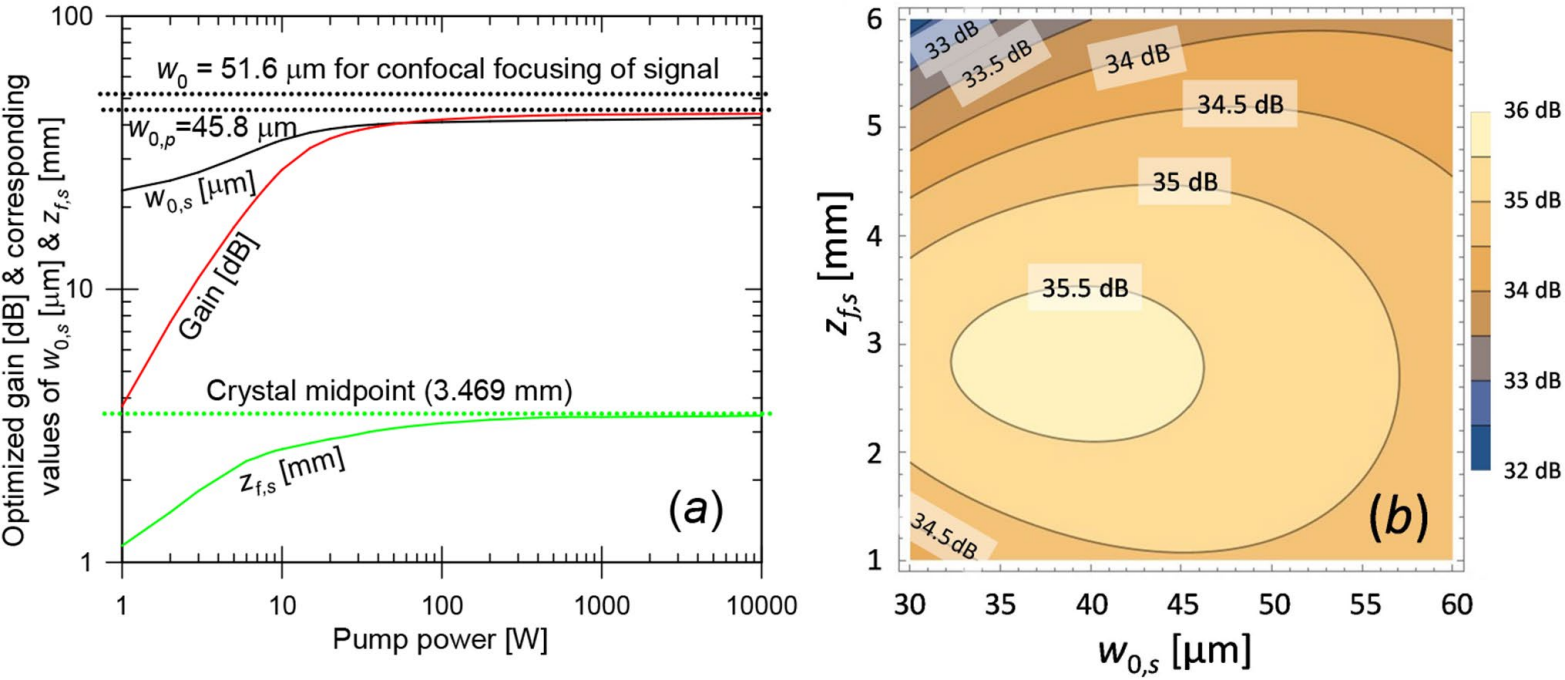
Case A, bulk crystal with confocal pump-focusing. (**a**) Optimized gain and the corresponding values of *w*_0,*s*_ and *z*_*f*,*s*_. The crystal midpoint and the beam waist radius corresponding to confocal focusing of the signal (51.6 μm) and the waist radius used for the pump (45.8 μm) are indicated, too. (**b**) contour plot of the gain vs. *w*_0,*s*_ and *z*_*f*,*s*_ at a pump power of 20 W

We first (Case A) examine pumping which is confocal (neglecting distortions). The undistorted pump waist radius *w*_0,*p*_ becomes 45.8 μm (Rayleigh length in crystal 8.5 mm). To illustrate the signal gain optimization needed for Eq. (2), Fig. 7 shows the optimized gain values and the corresponding values of *w*_0,*s*_ and *z*_*f*,*s*_. There is also a contour plot of the gain vs. *w*_0,*s*_ and *z*_*f*,*s*_ at a pump power of 20 W, for which the optimal values are *w*_0,*s*_ = 38.7 μm and *z*_*f*,*s*_ = 2.83 mm. The gain reaches 35.6 dB (*G*_*lin*_ = 3660). Since the pump decreases along the crystal, the optimal signal focus is in front of the pump focus.

**Fig. 8:**
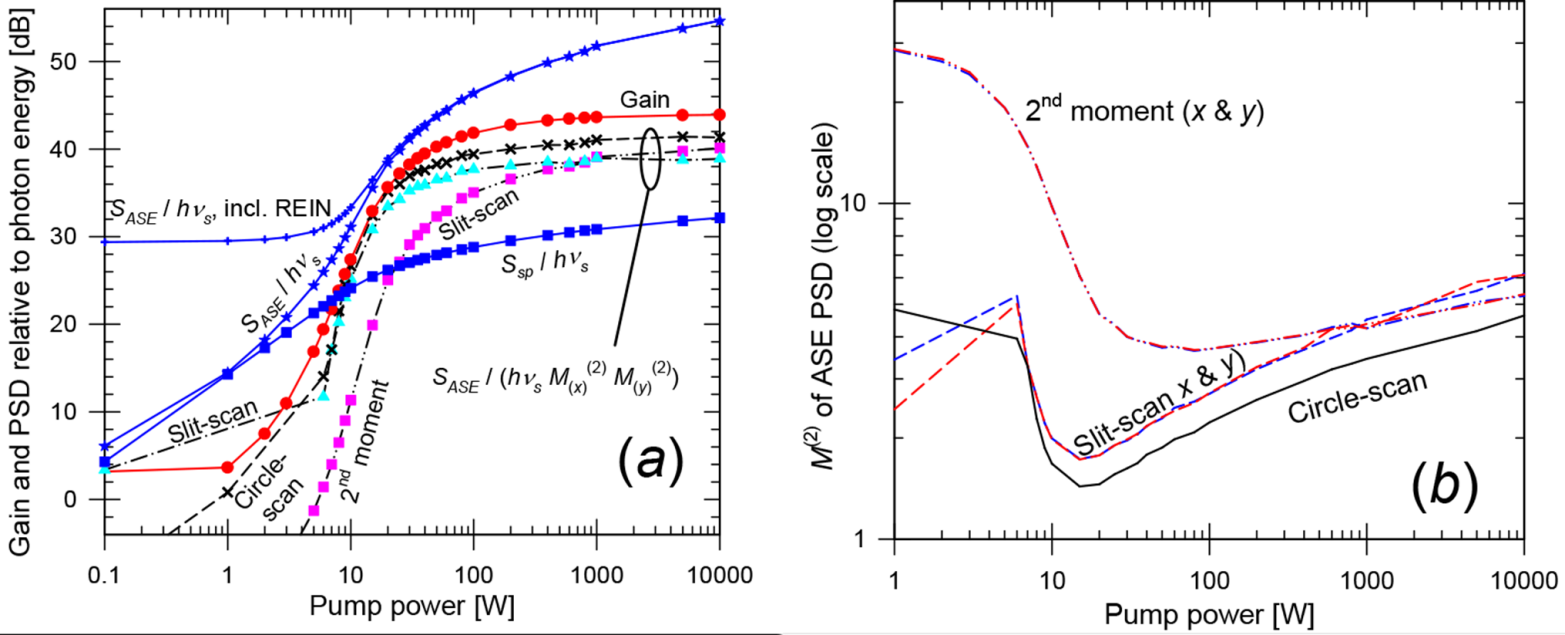
Case A, bulk crystal with confocal pump-focusing. (**a**) Gain, *S*_*sp*_ / *hν*, *S*_*ASE*_ / *hν* before and after REIN subtraction, and *S*_*ASE*_ / (*hν* $\:{{M}_{ASE}^{2}}^{2}$). (**b**) $\:{M}_{ASE}^{2}$ evaluated as a 2nd -moment and with slit-scanning (*x* and *y*), as well as with circle-scanning

Figure 8 presents the gain vs. pump power for the optimized *w*_0,*s*_ and *z*_*f*,*s*_. It also includes *S*_*ASE*_ and *S*_*ASE*_ / $\:{{M}_{ASE}^{2}}^{2}$ (both relative to the photon energy), and $\:{M}_{ASE}^{2}$ evaluated in different ways. The spontaneous-emission PSD *S*_*sp*_ at 2410 nm (in one polarization within the range of propagation angles supported by the BPM grid) is also plotted. This was evaluated as a spectral and angular fraction of the total fluorescence power. The BPM simulations used 200 longitudinal steps of 85 μm and 32 × 32 transverse gridpoints with a spacing of 16 μm (window size 512 μm). This supports propagation angles up to 30.7 mrad in the crystal for the signal and ASE. See Supplement 1 for a discussion of the parameters of the numerical grid. Circle-scanning yields the smallest deviations in Eq. (2), remaining below 3 dB for gain above ~ 20 dB. Notably, the ASE can be generated with high beam quality. With circle-scanning, the lowest $\:{M}_{ASE}^{2}$-value becomes 1.48 (pump power 15 W, gain 33 dB). At low pump power, *S*_*sp*_ agrees with *S*_*ASE*_ within 2 dB, thus dominating *S*_*ASE*_. The analytic calculation of *S*_*sp*_ neglects reabsorption, which partly explains why *S*_*sp*_ > *S*_*ASE*_ at low gain.

**Fig. 9:**
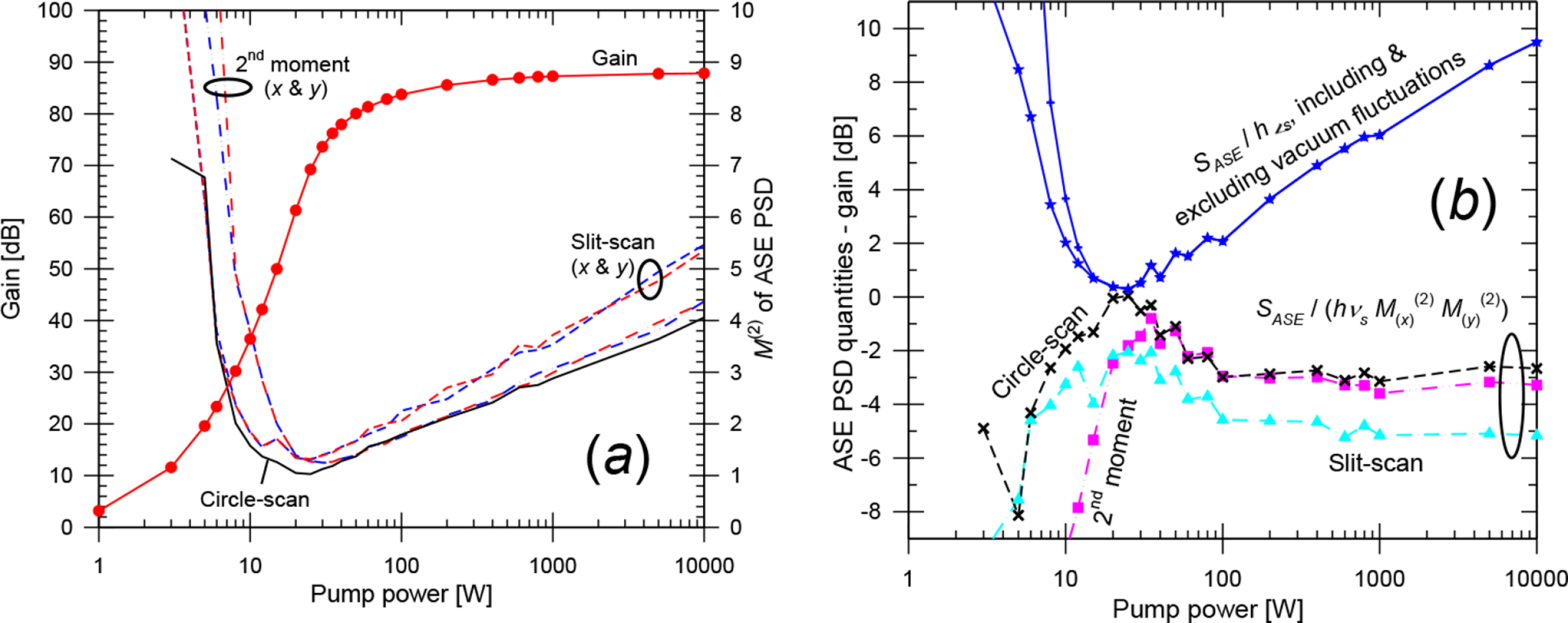
Case B, bulk crystal with confocal pump-focusing and double concentration. (**a**) Gain and $\:{M}_{ASE}^{2}$ evaluated as a 2nd -moment (*x* and *y*), with slit-scanning (*x* and *y*), and with circle-scanning. (**b**) ASE level *S*_*ASE*_ / *hν* (before and after REIN subtraction) and *S*_*ASE*_ / (*hν* $\:{{M}_{ASE}^{2}}^{2}$). $\:{M}_{ASE}^{2}$ is evaluated as indicated. $\:{{M}_{ASE}^{2}}^{2}$ & $\:{M}_{ASE,\:x}^{2}$ × $\:{M}_{ASE,\:y}^{2}$ are used equivalently. All curves are in dB, and the signal gain has been subtracted (e.g., (*S*_*ASE*_ / *hν*)_*dB*_ – gain in dB, etc.)

In Case A, the maximum gain is limited by the concentration–length product of the crystal. Case B is the same as Case A, except that the Cr^2+^-concentration is doubled to 14.05 × 10^24^ m^–3^. This allows for gain up to 87.9 dB at infinite pump power. Figure 9 shows the results. Due to the proximity of the quantities in Fig. 8 (a) when plotted on a scale capturing the full gain range, Fig. 9 (b) displays the differences between the gain and the quantities shown in Fig. 8 (a), while Fig. 9 (a) presents the gain and the $\:{M}_{ASE}^{2}$-values. The transverse numerical grid was the same as in Case A, while the step-length was halved to limit the gain in a step. Also as for Case A, the circle-scanned determination of $\:{M}_{ASE}^{2}$ fits best with Eq. (2). The deviations are below 3 dB for pump power > 8 W (gain > 30 dB). $\:{M}_{ASE}^{2}$ is lowest for 25 W of pump power (gain 69 dB), reaching 1.3 (slit-scan and 2nd -moment). With circle-scanning, $\:{M}_{ASE}^{2}$ becomes as low as 1.03 at 25 W of pump power, and the discrepancy in Eq. (2) becomes only 0.04 dB at that power.

When the total power in the full ASE spectrum is considered, ASE “self-saturation” (neglected in these simulations) becomes significant for pump powers in the range 15–5000 W, over which the gain exceeds 50 dB. See Supplement 1. This affects the accuracy of our simulations with narrowband ASE. However, neglecting self-saturation affects both sides of Eq. (2) and does not automatically invalidate a comparison between simulated ASE and gain, although it will impact comparisons between experiments and simulations.

**Fig. 10:**
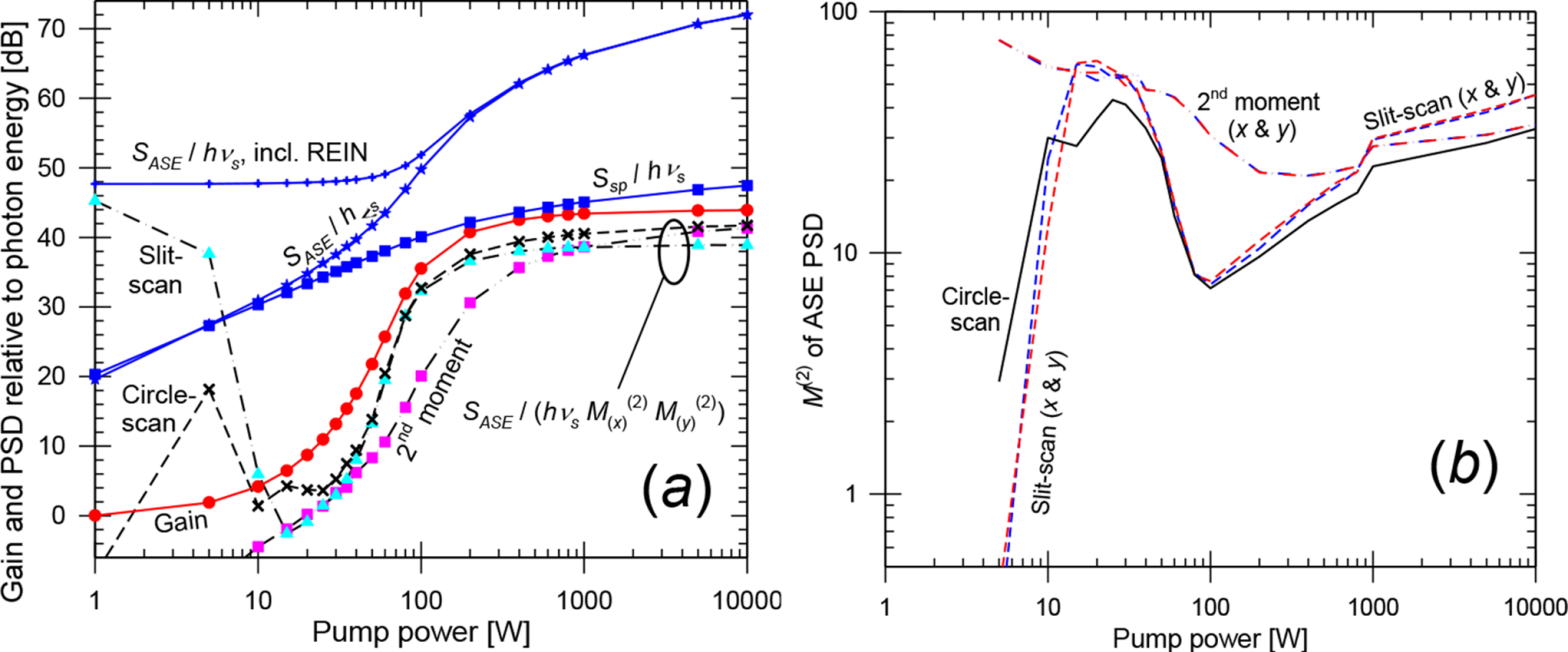
Case C, bulk crystal with *w*_0,*p*_ = 200 μm. (**a**) Gain, *S*_*sp*_ / *hν*, *S*_*ASE*_ / *hν* (before and after subtraction of residual equivalent input noise) and *S*_*ASE*_ / (*hν* $\:{{M}_{ASE}^{2}}^{2}$). (**b**) $\:{M}_{ASE}^{2}$ evaluated as a 2nd -moment (*x* and *y*), with slit-scanning (*x* and *y*), as well as with circle-scanning

Case C employs a much larger pump beam, *w*_0,*p*_ = 200 μm, for the original (default) concentration (Table 1). This leads to a much larger pump beam volume (1.07 mm^3^) than with confocal pumping (0.075 mm^3^) and therefore more ASE, e.g., *S*_*ASE*_ = 7.9 fW/Hz vs. 0.56 fW/Hz with confocal pumping at 35.5 dB of gain. The ASE simulations used 170 longitudinal steps of 100 μm and 256 × 256 transverse points with a spacing of 8 μm (half of Case A) for a window size of 2048 μm (four times that in Case A). This supports propagation angles up to 61.5 mrad at 2410 nm. Figure 10 shows the results. For the circle-scan, the quantity *S*_*ASE*_ / $\:{{M}_{ASE}^{2}}^{2}$ is within 3 dB of the achievable gain for gain > 33 dB (pump power > 85 W). In this range, *S*_*ASE*_ is over 8 dB larger than *S*_*sp*_. Compared to confocal pumping, the ASE beam propagation factor $\:{M}_{ASE}^{2}$ is considerably worse in Case C. With circle-scanning, it reaches a minimum of 7.1 for a pump power of 100 W (gain 35.5 dB).

**Fig. 11:**
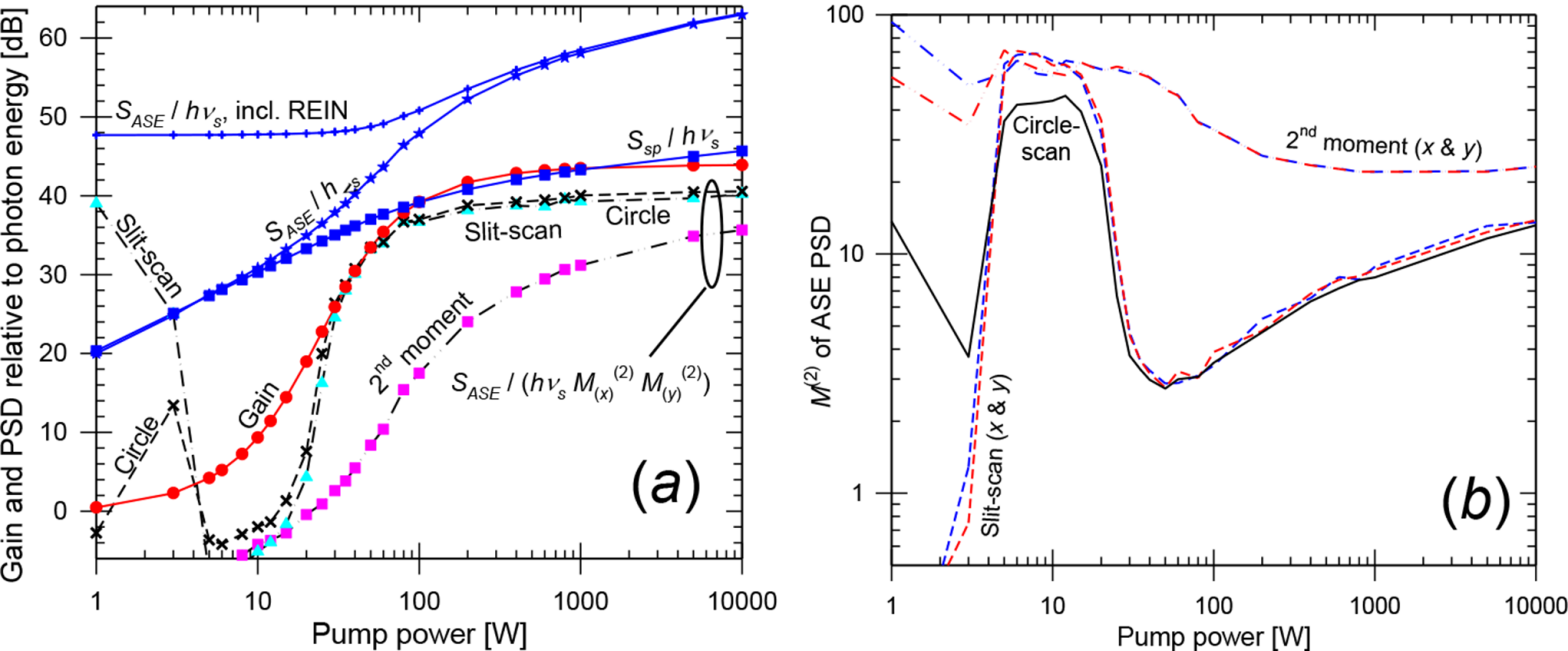
Case D, bulk crystal with *w*_0,*p*_ = 8 μm. (**a**) Gain, *S*_*sp*_ / *hν*, *S*_*ASE*_ / *hν* (before and after subtraction of residual equivalent input noise) and *S*_*ASE*_ / (*hν* $\:{{M}_{ASE}^{2}}^{2}$). (**b**) $\:{M}_{ASE}^{2}$ evaluated as a 2nd -moment (*x* and *y*) and with slit-scanning (*x* and *y*), as well as with circle-scanning

Case D, finally, considers tight focusing of the pump, *w*_0,*p*_ = 8 μm. Because of the rapid diffraction (Rayleigh length 0.26 mm in the crystal), the pump beam volume is larger than for the confocal case, 0.61 mm^3^. The numerical grid was the same as in Case C. Figure 11 shows the results. With circle-scanning, the quantity *S*_*ASE*_ / $\:{{M}_{ASE}^{2}}^{2}$ is within 3.7 dB of the achievable gain for gain > 22 dB (pump power > 24 W). In this range, *S*_*ASE*_ is at least 2 dB larger than *S*_*sp*_. Again, the deviation from Eq. (2) is larger for the other methods of determining $\:{M}_{ASE}^{2}$ (especially 2nd -moment). The beam propagation factor $\:{M}_{ASE}^{2}$ (circle-scan) reaches a minimum of 2.7 for 50 W of pump power (gain 33.4 dB).

## Discussion

In the cases we have examined, the BPM-simulations with equivalent input noise followed by subtraction of the residual equivalent input noise are accurate for the power spectral density *S*_*ASE*_, not only at high gain when spontaneous emission (*S*_*sp*_) is negligible but also at low gain when it dominates. Specifically, for the single-mode fiber, *S*_*ASE*_ as calculated with BPM agrees with that of the well-proven MPE approach for gains above ~ 24 dB (Fig. 2). At lower gain, the numerous modes supported by the BPM grid means that more of the spontaneous emission is captured than with the MPE approach, which only treats the guided modes. Therefore, although *S*_*ASE*_ as calculated with the two methods will differ, we still expect *S*_*ASE, BPM*_ – *S*_*sp*_ ≈ *S*_*ASE, MPE*_ at low gain. We verified this to be the case for the single-mode fiber, and thus the accuracy of the BPM-approach in this regime, with only a small deviation at the few-photon level.

The accuracy of the subtraction of the residual equivalent input noise is noteworthy. Our results demonstrate its accuracy down to the few-photon level, even when the equivalent input noise comprises 65,536 photons. This accuracy was obtained with ensemble-averaging of, e.g., 4000 realizations used at low gain, and is consistent with a standard deviation of (65,536 / 4000)^1/2^ = 4.0 photons. This corresponds to − 42.1 dB of 65,536 photons. For a single mode with equivalent input noise of one photon, the standard deviation of the averaged value becomes 4000^–1/2^ = 0.016 photons or − 18.0 dB. The standard deviation is unaffected by the REIN subtraction, which is deterministic.

This high accuracy justifies our approach for evaluating and subtracting the REIN.

The observations are similar for the multimode fiber in Fig. 5. For instance, BPM and MPE-calculations of *S*_*ASE*_ agree well for gains larger than ~ 27 dB. On the other hand, as the gain decreases (corresponding to shorter fibers), the agreement between *S*_*ASE*_ and analytic calculations of *S*_*sp*_ becomes worse than for the single-mode fiber, e.g., at 10 and 15 mm. The agreement then improves again for fibers shorter than 5 mm (gain up to ~ 6 dB). One possible explanation for this observation is that at the higher gains of 10 and 15-mm fibers, the large core leads to significant amplification of unguided light in the BPM simulations. It is important to note that this does not imply that the calculation of *S*_*ASE*_ is less accurate, even if the definition of the regime where spontaneous emission dominates becomes less distinct in these scenarios.

MPE-simulations are not available for the bulk amplifier, but we can still assess the agreement between BPM-calculations of *S*_*ASE*_ at low gain (when it is dominated by spontaneous emission) and *S*_*sp*_ as follows. We determined *S*_*sp*_ from the pump power absorbed in the BPM-calculations, assuming unity quantum efficiency and with negligible reabsorption and stimulated emission due to the signal and ASE (within 1 Hz). Calculations on Case A (not shown in Fig. 5 (a)) revealed that for low gain, these agreed within a fraction of a photon, even when the REIN subtraction reduced *S*_*ASE*_ by over 99.9%, to ~ 0.4 photons. In this case, the BPM grid comprised 32 × 32 points, resulting in a standard deviation of (32 × 32 / 4000)^1/2^ = 0.51 photons in the ensemble-averaged equivalent input noise. For Case C, *S*_*ASE*_ agreed with *S*_*sp*_ within 2 dB for gain below 11 dB (pump power < 25 W). For Case D, *S*_*ASE*_ agreed with *S*_*sp*_ within 2 dB for gain below ~ 20 dB (pump power < 22 W).

We do not present any experimental results, but note that for comparisons to experiments, a square aperture can be used in the farfield to reject light at angles not supported by the BPM grid. This may not be necessary if *S*_*ASE*_ forms a well-defined beam within the BPM grid, but some assessment of the capture is required, since the spontaneous emission can be quite significant also at large angles. If *S*_*ASE*_ does not form a beam of sufficient definition for nearly lossless or calibrated coupling into an optical spectrum analyzer (OSA) for direct measurement of *S*_*ASE*_ then one can instead measure the shape of the full ASE spectrum with an OSA and the total ASE power with a power meter and determine *S*_*ASE*_ from that. For this, the effective ASE bandwidth as calculated in Supplement 1 can be used. Additionally, more precise modeling can be considered, e.g., to account for the coupling of the emission into a collection fiber or the transverse boundaries of the gain fiber or crystal. If needed, these approaches can provide a more comprehensive and accurate comparison. The beam propagation factors $\:{M}_{ASE}^{2}$ calculated from the spatial distribution of the simulated ASE (farfield, exit-plane, and / or some other plane or planes) can also be compared to experimental values, preferably determined in the same way (e.g., slit-scan) and without any mismatch in the captured propagation angles to reduce the sources of discrepancy.

Our simulations show that Eq. (2) can determine the gain from *S*_*ASE*_ and $\:{M}_{ASE}^{2}$ with reasonable accuracy in some, but not all, circumstances. We would like to identify characteristics that gauge the validity of Eq. (2) with parameters that are straightforward to measure. We reiterate that the gain *G*_*lin*_ in Eq. (2) is the highest achievable gain for a specific combination of crystal, pump focusing, and pump power. Furthermore, Eq. (2) relies on $\:{{M}_{ASE}^{2}}^{2}$ corresponding to an effective number of modes. We first point out that this cannot always hold. A counterexample is a pump comprising two parallel beams. Each of these can generate a (nearly) diffraction-limited ASE-beam, thus they comprise approximately two modes combined. However, the *M*^2^-value of the combined beams depends on their separation and can be arbitrarily large. A ring-shaped pump beam is a similar counterexample. Still, $\:{{M}_{ASE}^{2}}^{2}$ may be a reasonable approximation for the effective number of modes in our simulations, in which the transverse gain profiles are largely convex without significant dips. We restrict this discussion to circle-scanned $\:{M}_{ASE}^{2}$-values determined at the 1/*e*^2^ intensity level, as this method worked best for Eq. (2).

**Fig. 12:**
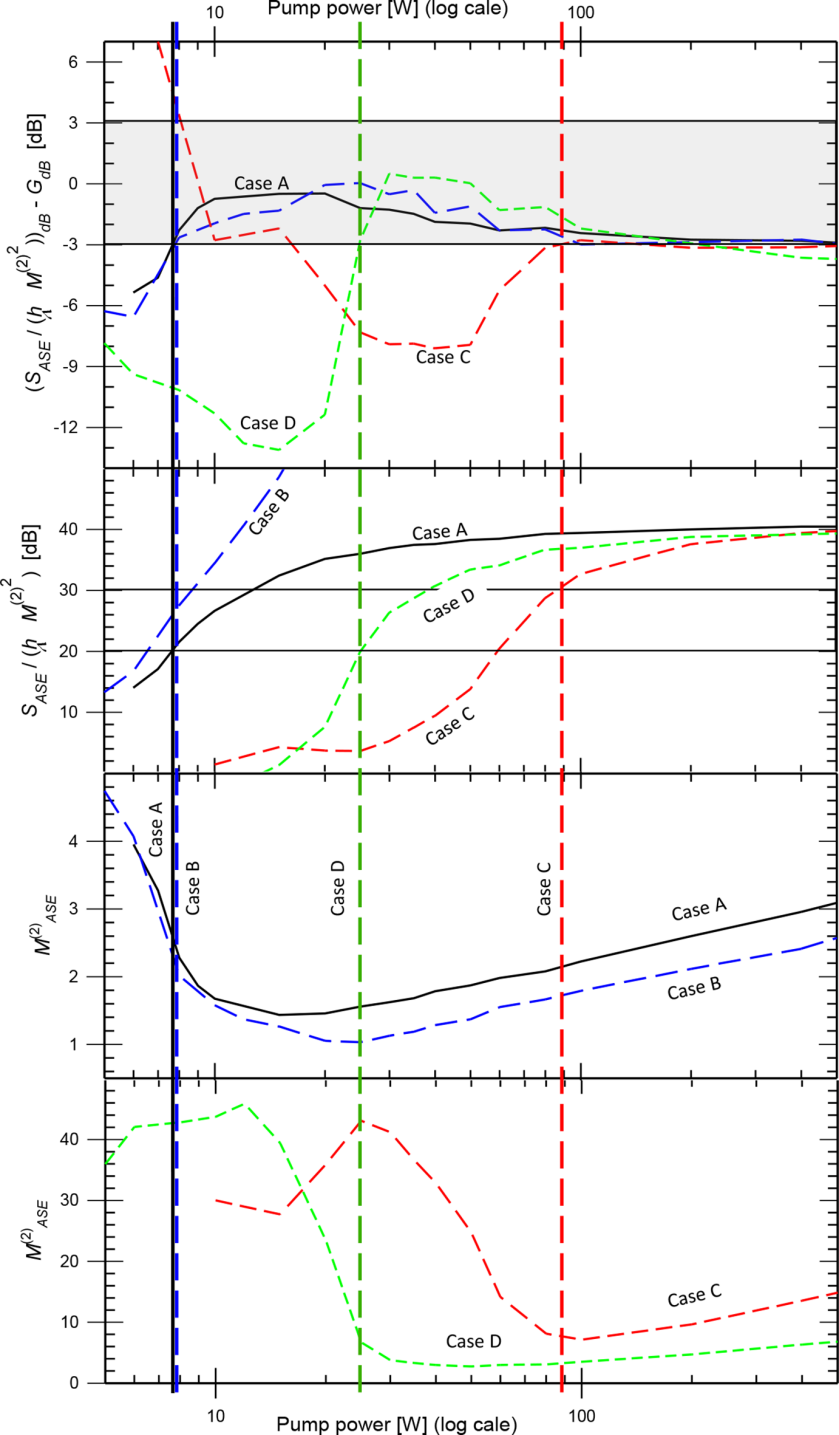
Discrepancy in Eq. (2) vs. pump power for cases A – D (top) plotted together with *S*_*ASE*_ / (*hν* $\:{{M}_{ASE}^{2}}^{2}$) and $\:{M}_{ASE}^{2}$. The vertical lines separate regions with large and small discrepancy in Eq. (2) for the four cases and the shaded area in the top graph indicates the range for good agreement, defined as ± 3 dB

The most obvious gauge is that at high gain, the adherence to Eq. (2) is fair or good. In the four cases we studied, the discrepancy in Eq. (2) became smaller than ~ 3 dB for gain above 20–30 dB. However, this gauge requires that the optimal gain is at least 20–30 dB, which may be difficult to know. We would rather gauge the validity from *S*_*ASE*_ and $\:{M}_{ASE}^{2}$, which would anyway be calculated or measured. Figure 12 shows the discrepancy in Eq. (2) vs. pump power for the four bulk amplifier cases, as well as *S*_*ASE*_ / (*hν* $\:{{M}_{ASE}^{2}}^{2}$) and $\:{M}_{ASE}^{2}$. The vertical lines indicate transition points between large and small discrepancy. Although somewhat arbitrarily defined, we see that these transitions occur when *S*_*ASE*_ / (*hν* $\:{{M}_{ASE}^{2}}^{2}$) is in the range 20–30 dB. Furthermore, the transitions correlate with sharp increases in $\:{M}_{ASE}^{2}$ towards lower pump power.

These criteria have significant variations and gray zones, and the $\:{M}_{ASE}^{2}$-criterion furthermore requires knowledge of $\:{M}_{ASE}^{2}$ for a range of pump powers. We were not able to identify a better criterion but will discuss some of the options we investigated.

**Fig. 13:**
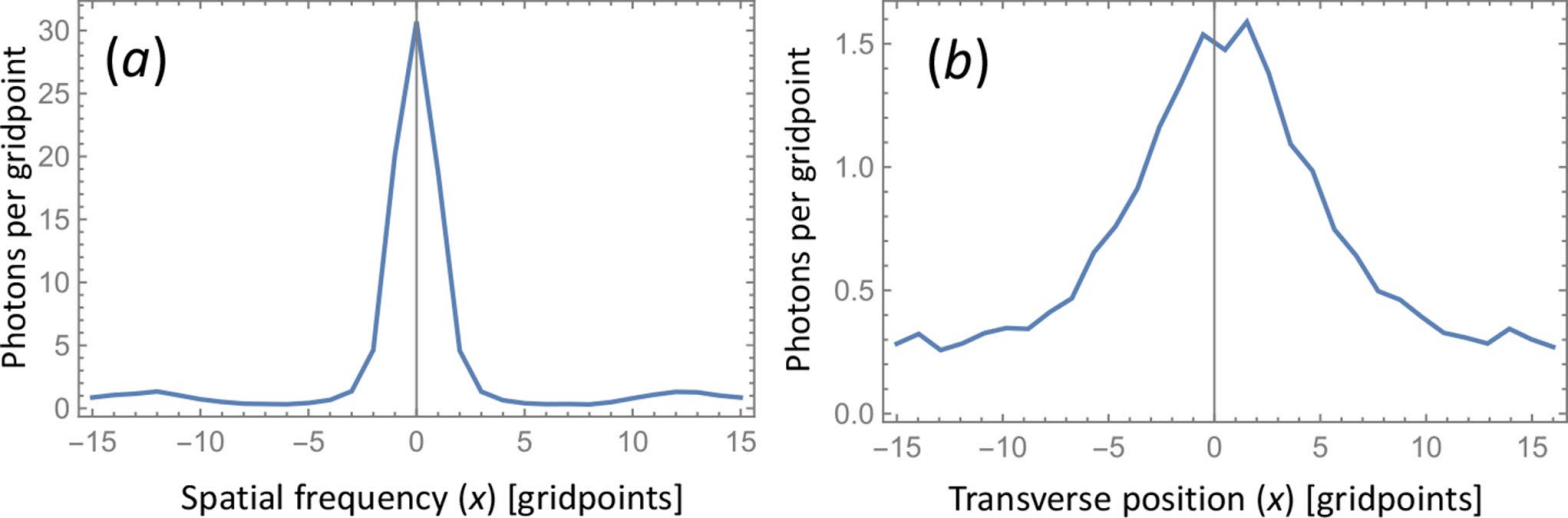
(**a**) Farfield and (**b**) exit-plane distribution of *S*_*ASE*_ along a line through the center of the beam for Case A at 6 W of pump power

The correlation with the beam quality suggests that the formation of a beam of ASE could be a criterion. This would be easy to determine with a camera in the farfield. However, this criterion did not work. Figure 13 (a) shows a cut through the center of the ASE beam in the farfield for Case A at 6 W of pump power. A lens at the exit plane minimized the divergence. The peak is ~ 16 dB above the background, but still, the discrepancy in Eq. (2) is 5.4 dB. The exit-plane distribution has a peak ~ 8 dB above the background (Fig. 13 (b)) and is thus less pronounced than the peak in the farfield. This may explain why a sharp beam (i.e., farfield peak) does not guarantee good adherence to Eq. (2).

The appropriateness of a configuration could also constitute a gauge for the validity of Eq. (2). Thus, the discrepancy in Eq. (2) may be large only for unreasonable configurations or parameters of little interest, such as those with very low gain or unrealistically high pump power. For instance, Case D has pump focusing that is far from optimal. (Case C may be more relevant insofar as it resembles highly multimode pumping.) Confocal pumping is not optimal, either, but is close. Furthermore, in terms of optimization, we note that the crystal length relates not only to the focusing but also the power of the pump, i.e., for a given pump power, the crystal length is optimal when the leaked pump is just enough to excite enough ions to create gain at the output end of the crystal. For Case A, this match between crystal length and pump power occurs for 6 W of pump power, which is thus highly relevant. However, the discrepancy in Eq. (2) is significant at that power. In Case B (confocal pumping with twice the concentration), the similarly matching pump power becomes ~ 16.6 W, and now, the discrepancy in Eq. (2) is small. We attribute the improved agreement to the higher gain in Case B, ~ 53.7 dB at 16.6 W of pumping, compared to 19 dB (at 6 W of pumping) in Case A. We conclude that a pump with focusing and power well-matched to a specific crystal is not enough to ensure the validity of Eq. (2).

We noted previously that the larger pump volume of Case C and D increases the ASE for a given signal gain. This is reflected by the difference between the curves for the signal gain and for *S*_*ASE*_ / *hν*_*s*_ in Fig. 10 (a) and Fig. 11 (a). See also Eq. (1). The pumping is far from optimal in those cases. Improved pump parameters lead to higher signal gain for a given pump power, and reduced difference between those curves. Thus, the difference is smaller in Case A with confocal pumping (Fig. 8 (a)), ~ 3 dB or less for pump powers in the range 10–35 W, with signal gain 28–37 dB. For other pump powers, the ASE PSD is more than 3 dB higher than that suggested by Eq. (1). For example, it reaches ~ 6 dB for a pump power of 250 W. This excess ASE may be a result of a pump intensity that is sufficiently high to create significant gain also in the wings of the Gaussian pump beam, thus increasing the gain volume beyond the diffraction limit. Note also that although the confocal pumping we used is close to optimal, fully optimized pump beam and crystal parameters may further reduce the ASE PSD for a specific signal gain (in addition to reducing the required pump power). It is also clear that to minimize the ASE for a given signal gain, the signal should be at the gain peak.

Case B also uses confocal pumping, but with twice the concentration as in case A. Again, the deviation from Eq. (1) is small over a range of pump powers, and is less than 1 dB for a pump power of 15 W. This suggests that the ASE is close to single-moded at this wavelength. The signal gain of 50 dB is at the limit of where we consider ASE self-saturation to be negligible. Note also that the ASE beam quality may be significantly worse at other wavelengths where the gain is lower. This can affect the beam quality of the ASE as a whole, although the effect may be small since at high gain, the ASE PSD is strongly peaked at the gain peak. This is straightforward to simulate with our approach. We also note that the backward ASE will differ, and this could be significant. However, these points were not investigated.

Our BPM-simulations assume that the equivalent input noise consists of one photon per gridpoint, corresponding to *n*_*sp*_ = 1, but any background loss or ground-state absorption of signal photons makes *n*_*sp*_ larger and dependent on the fractional excitation *n*_2_. See Eq. (3). Yet, in the absence of ASE self-saturation, the simulations are linear in *n*_*sp*,_ so our assessment of Eq (2) is equally valid for any value of *n*_*sp*_, if it is the same in each gridpoint. Therefore, precise accuracy in *n*_*sp*_ is not crucial in this regard, when we are not comparing to experiments. However, *n*_2_ varies across the input grid, with *n*_2_ ≈ 0 at the unpumped edge of the window. The simulations do not include any background loss, but there is some ground-state absorption. Therefore, Eq. (3) suggests that a constant value of *n*_*sp*_ across the input grid is not the best choice. We have not tried to determine better, potentially position-dependent, values for *n*_*sp*_. However, to estimate the errors resulting from the use of a constant *n*_*sp*_ across the full input grid, we compared results of Case A with 50 W of pump power and *n*_*sp*_ = 1 everywhere to those where the noise seeding was restricted to the effectively pumped parts of the input grid, and *n*_*sp*_ = 0 elsewhere. The difference incurred by this spatial variation of *n*_*sp*_ was negligible, indicating that constant seeding across the window with *n*_*sp*_ = 1 is a reasonable assumption in this simulation. The validity of uniform seeding is further supported by the good agreement with spontaneous-emission calculations. However, we have not explored what happens when there is significant ground-state absorption of the signal. Then, distributed seeding of the ASE (by spontaneous emission) [7, 17, 19, 20] may be a better choice, since this avoids the problem of spatial variations in *n*_*sp*_ in the input grid. It is unclear how a spatially varying value of *n*_*sp*_ would be determined, given also that more precise determinations of *n*_*sp*_ depend on the longitudinal distribution of the gain and ground-state absorption (e.g., [7]). Distributed seeding also avoids the need for subtraction of residual equivalent input noise (although this is quite accurate in our simulations).

The Petermann *K*-factor *K* [5, 6, 8, 9] may play a role in the discrepancy observed in our simulations in relation to Eq. (2). Given that *K* ≥ 1, this can lead to a higher ASE-power in a mode than Eqs. (1) and (2) suggest. However, the effective number of modes $\:{{M}_{ASE}^{2}}^{2}$ is generally larger than *S*_*ASE*_ and *G*_*lin*_ suggests according to Eq. (2). See Fig. 12 (top) as well as Fig. 5 (a), 8 (a), 9 (b), 10 (a), and 11 (a). A value *K* > 1 would increase the ASE per mode and thus exacerbate this discrepancy (as does *n*_*sp*_ > 1).

Instead of evaluating *K*, we view this as a factor that can affect the observed discrepancy.

We next discuss if our neglect of ASE self-saturation is justified (irrespective of the accuracy of Eq. (2)). In the absence of self-saturation, it is possible to simulate only the small part of the ASE power that lies within 1 Hz, and to disregard counter-propagating ASE. By contrast, in the presence of self-saturation, accurate simulations must consider the ASE in both directions and include the total ASE power *P*_*ASE*_ in both polarizations in the full spectrum. For ASE counter-directional to the signal, the ASE seeding occurs in the signal output end, so *n*_*sp*_ needs to be evaluated in that end. However, given also that convergence and the number of iterations required are concerns with contra-directional saturating waves (e.g., [19]), this considerably more demanding calculation is beyond our scope. Instead, to assess if ASE self-saturation can be neglected, we simulated *P*_*ASE*_ in the same way as we did *S*_*ASE*_ at 2410 nm, i.e., with a monochromatic wave, but with the equivalent-input noise seeding scaled by an effective ASE bandwidth Δ*ν*_*eff*_ and the number of polarizations (= 2). The effective bandwidth depends on the gain and was calculated from a spectral integral of Eq. (1), i.e., the total ASE power in a single mode. See Supplement 1 for details. For the crystal of Case A, C, D, the effective linewidth varies from 35.6 THz (690 nm) at low gain to 14.6 THz (282 nm) at 44.0 dB (the highest that can be reached with 1901-nm pumping). The saturation (i.e., compression) of the gain at 2410 nm was < 0.05 dB in Case A, C, D, with forward-propagating ASE only. Even if the compression approximately doubles with bidirectional ASE, this can still be neglected.

By contrast, Case B allows for higher gain, and self-saturation can become significant for gain exceeding 50 dB. Note however that this does not automatically invalidate Eq. (2), and the validation of the simulation approach remains valid in the sense that it is based on comparisons of different simulated parameters that all derive from the same spatial gain distribution and are thus all affected by the inclusion or neglect of self-saturation. Consequently, Eq. (2) can remain valid for simulated quantities even when significant saturation is neglected. Likewise, it may be possible to use experimental values of $\:{M}_{ASE}^{2}$ and *S*_*ASE*_ to calculate the achievable gain according to Eq. (2), insofar as the saturated transverse gain profile does not cause problems and the limits of validity discussed above are observed. However, simulations and experiments would not agree.

Whereas *S*_*ASE*_ (in 1 Hz) is easier to simulate, the power in the whole ASE spectrum, *P*_*ASE*_, is easier to measure. Therefore, we estimated the relation between *S*_*ASE*_ (at 2410 nm) and *P*_*ASE*_ in the unsaturated regime. We found that *P*_*ASE*_ ≈ (*S*_*ASE*_ / *hν*) × 2.5 µW for gain up to 40 dB in the crystal of Case A, C, D. For instance, with four effective modes ($\:{M}_{ASE}^{2}$ = 2) per polarization, 20 dB of gain can be expected to generate 2 mW of forward ASE power. See Supplement 1 for details.

Our signal power was sufficiently low to avoid saturation, and the accuracy of our approach for a stronger, saturating, signal would have to be investigated. A saturating signal co-propagating with the pump is straightforward to simulate, but spatial hole-burning may cause the transverse gain profile to become concave. This can invalidate Eq. (2). Supplement 1 provides further details and discussions on the calculation of ASE, effective bandwidth, ASE self-saturation, and *n*_*sp*_.

## Conclusions

We have investigated and used the beam propagation method with equivalent input noise for the simulation of narrow-band amplified spontaneous emission at the signal wavelength and signal amplification in continuous-wave Cr^2+^:ZnSe non-waveguiding “bulk” amplifiers with non-saturating signal and ASE in different configurations. Both the incident pump at 1901 nm and the signal at 2410 nm were diffraction-limited gaussian beams. The signal wavelength coincided with the peak of the gain, which was homogenously broadened. The absorption at the signal wavelength in the un-pumped crystals was between 0.48 dB and 0.95 dB, so any reabsorption at weak pumping was small. We implemented the equivalent input noise as random realizations of one photon per gridpoint (corresponding to *n*_*sp*_ = 1), and we showed that this leads to the familiar one noise photon per mode. We conducted between 100 and 6000 simulations with different realizations of the random input noise, then ensemble-averaged the outcomes to determine the power spectral density of the ASE, including its spatial distribution in the exit-plane and farfield. Our approach is compatible with standard BPM code, as long as the equivalent input noise is correctly implemented. While it is not necessary to define any modes, we validated the simulations of the ASE PSD by comparing results for single-mode and multimode fiber amplifiers to those obtained with well-established conventional fiber amplifier models.

We also calculated the beam quality of the ASE at the signal wavelength, $\:{M}_{ASE}^{2}$, with different methods. We investigated if $\:{{M}_{ASE}^{2}}^{2}$ could serve as an estimate of an effective number of ASE modes and, when coupled with the ASE PSD, predict achievable signal gain. Notably, the value of $\:{M}_{ASE}^{2}$ varied considerable between the different methods. Depending on the amplifier configuration, we found that at gain higher than 20–30 dB it became possible to determine the achievable signal gain from the ASE characteristics with reasonable accuracy. It is also possible to evaluate the spontaneous emission, and we found agreement down to the single-photon level. We note that the ASE beam propagation and $\:{M}_{ASE}^{2}$ depends on the wavelength and can be different for the full ASE spectrum. We did not investigate thermo-optic effects, nor cases with strong reabsorption or saturation (e.g., strong bidirectional ASE spectra). Even if seeding with equivalent input noise remains valid, the modification of the numerical solver to treat such effects would require significantly more work.

## Electronic supplementary material

Below is the link to the electronic supplementary material.

Supplementary Material 1

## Data Availability

Data supporting this study are openly available from the University of Southampton repository at 10.5258/SOTON/D3313 [29].

## Appendix 1

We show that the assumption of one (or *n*_*sp*_) noise photon per gridpoint in the BPM input grid leads to the excitation of one (or *n*_*sp*_) noise photon per mode. We use the convention that the optical intensity *I* (W/m^2^) of a lightwave is related to the complex field according to *I* (*x*, *y*, *z*) = |*A* (*x*, *y*, *z*)|^2^, where *x* and *y* are transverse coordinates and *z* is the longitudinal coordinate. The power of a paraxial lightwave in a transverse plane *z* becomes,

4 $$\:P=\iint\:I\:\left(x,\:y,\:z\right)\:dxdy=\iint\:{\left|A\:\left(x,\:y,\:z\right)\right|}^{2}dxdy$$

We further introduce modal fields *A*^*mf*^ (*x*, *y*, *z*) and normalize them according to

5 $$\:\iint\:{\left|{A}^{mf}\:\left(x,\:y,\:z\right)\right|}^{2}dxdy=1\:W$$

Also, let *A*_*m*_ be a modal amplitude (complex and dimensionless) so that the complex field of an excited mode is given by *A* (*x*, *y*, *z*) = *A*_*m*_ *A*^*mf*^ (*x*, *y*, *z*). The power *P*_*m*_ of a mode becomes *P*_*m*_ = 1 W × |*A*_*m*_|^2^. When excited by an external field *A*, *A*_*m*_ becomes,

6 $$\:{A}_{m}={\left(1\:W\right)}^{-1}\iint\:{A}^{*}\left(x,\:y,\:z\right)\:{A}^{mf}\left(x,\:y,\:z\right)\:dxdy$$

where the star denotes complex conjugation. See, e.g. [28], for details on modes and their excitation.

On an equidistant discrete grid such as that we use in BPM, the integrals are replaced by sums, e.g.,

7 $$\:\sum\limits_{i,\:j}{\left|{A}^{mf}\:\left({x}_{i},\:{y}_{j},\:z\right)\right|}^{2}{\Delta\:}x\:{\Delta\:}y={\Delta\:}x\:{\Delta\:}y\sum\limits_{i,\:j}{\left|{A}_{ij}^{mf}\right|}^{2}=1\:W\:$$

8 $$\begin{aligned}{A}_{m}&={\left(1\:W\right)}^{-1}\:{\Delta\:}x\:{\Delta\:}y\:\sum\limits_{i,\:j}{A}_{ij}^{*}{A}_{ij}^{mf}\\&={\left(1\:W\right)}^{-1}\:{\Delta\:}x\:{\Delta\:}y\:\sum\limits_{i,\:j}{A}_{ij}^{*}\left|{A}_{ij}^{mf}\right|{e}^{i{\varphi\:}_{ij}^{mf}}\end{aligned}$$

9 $$\:P={\Delta\:}x\:{\Delta\:}y\sum\limits_{i,\:j}{\left|{A}_{ij}\right|}^{2}\:$$

where Δ*x* and Δ*y* are the grid spacings and (*x*_*i*_, *y*_*j*_) are the coordinates of gridpoint (*i*, *j*). We also adopt the convention $\:{A}^{mf}\:\left({x}_{i},\:{y}_{j},\:z\right)={A}_{ij}^{mf}$ (and similarly for other quantities) and for simplicity drop the explicit *z*-dependence of the fields. In Eq. (8), we have also written $\:{A}_{ij}^{mf}$ on its polar form, $\:\left|{A}_{ij}^{mf}\right|{e}^{i{\varphi\:}_{ij}^{mf}}$.

To proceed, we let *A*_*ij*_ represent an input noise field comprising normally-distributed random complex variables with zero mean and circular symmetry so that the distributions of the real and imaginary parts are the same and independent. From Eq. (8) the expectation value of the modal excitation becomes

10 $$\begin{aligned}E\left[{A}_{m}\right]&=E\left[{\left(1\:W\right)}^{-1}{\Delta\:}x\:{\Delta\:}y\:\sum\limits_{i,\:j}{A}_{ij}^{*}{A}_{ij}^{mf}\right]\\&={\left(1\:W\right)}^{-1}{\Delta\:}x\:{\Delta\:}y\:\sum\limits_{i,\:j}E\left[{A}_{ij}^{*}\right]{A}_{ij}^{mf}=0\end{aligned}$$

*E* [*] denotes expectation values, which we evaluate as ensemble-averages in our BPM-simulations. Furthermore,

11 $$\begin{aligned}E\left[{\left|{A}_{m}\right|}^{2}\right]&=\:E\left[{\left|{\left(1\:W\right)}^{-1\:}{\Delta\:}x\:{\Delta\:}y\:\sum\limits_{i,\:j}{A}_{ij}^{*}{A}_{ij}^{mf}\right|}^{2}\right]\\&={{\left(1\:W\right)}^{-2\:}\left({\Delta\:}x\:{\Delta\:}y\right)}^{2}\:E\left[{\left|\:\sum\limits_{i,\:j}{A}_{ij}^{*}{A}_{ij}^{mf}\right|}^{2}\right]\\&={{\left(1\:W\right)}^{-2}\:\left({\Delta\:}x\:{\Delta\:}y\right)}^{2}\:E\left[{\left|\:\sum\limits_{i,\:j}{A}_{ij}^{*}\left|{A}_{ij}^{mf}\right|{e}^{i{\varphi\:}_{ij}^{mf}}\right|}^{2}\right]\\&={{\left(1\:W\right)}^{-2}\:\left({\Delta\:}x\:{\Delta\:}y\right)}^{2}\:E\left[{\left|\:\sum\limits_{i,\:j}{A}_{ij}\left|{A}_{ij}^{mf}\right|\right|}^{2}\right]\end{aligned}$$

For the last equality in Eq. (11), we have used that a distribution which is circularly symmetric in the complex plane is independent of the phase argument and therefore its expectation value does not change when multiplied by the phase factor $\:{e}^{i{\varphi\:}_{ij}^{mf}}$.

We next substitute *A*_*ij*_ by (*P*_*ij*_^*sample*^ / Δ*x* Δ*y*)^1/2^ (*ξ*_*ij*_ + *i η*_*ij*_), where *P*_*ij*_^*sample*^ is the expectation value of the sample power (= *E* [|*A*_*ij*_|^2^] Δ*x* Δ*y*) and *ξ*_*ij*_ and *η*_*ij*_ are real-valued normally distributed random variables. From our assumptions, it follows that they have zero mean and the same variance. It also follows that *E* [|*ξ*_*ij*_ + *i η*_*ij*_|^2^] = *E* [*ξ*_*ij*_^2^] + *E* [*η*_*ij*_^2^] = 1, so *E* [*ξ*_*ij*_^2^] = *E* [*η*_*ij*_^2^] = 1/2. We can then write Eq. (11) as

12 $$\begin{aligned}E\:\left[{\left|{A}_{m}\right|}^{2}\right]&={{\left(1\:W\right)}^{-2}\:\left({\Delta\:}x\:{\Delta\:}y\right)}^{2}\:E\:\left[{\left|\:\sum\limits_{i,\:j}{A}_{ij}\left|{A}_{ij}^{mf}\right|\right|}^{2}\right]\\&={\left(1\:W\right)}^{-2}\:{\Delta\:}x\:{\Delta\:}y\:{P}^{sample}\\&E\:\left[{\left|\:\sum\limits_{i,\:j}\left({\xi\:}_{ij}+i{\:\eta\:}_{ij}\right)\left|{A}_{ij}^{mf}\right|\right|}^{2}\right]\\&=\:2\:{\left(1\:W\right)}^{-2}\:{\Delta\:}x\:{\Delta\:}y\:{P}^{sample}\\&E\:\left[{\left|\:\sum\limits_{i,\:j}{\xi\:}_{ij}\left|{A}_{ij}^{mf}\right|\right|}^{2}\right]\\&=2\:{\left(1\:W\right)}^{-2}\:{\Delta\:}x\:{\Delta\:}y\:{P}^{sample}\\&E\:\left[{\left(\:\sum\limits_{i,\:j}{\xi\:}_{ij}\left|{A}_{ij}^{mf}\right|\right)}^{2}\right]\\&=2\:{\left(1\:W\right)}^{-2}\:{\Delta\:}x\:{\Delta\:}y\:{P}^{sample}\:Var\:\left[\sum\limits_{i,\:j}{\xi\:}_{ij}\left|{A}_{ij}^{mf}\right|\right]\\&=2\:{\left(1\:W\right)}^{-2}\:{\Delta\:}x\:{\Delta\:}y\:{P}^{sample}\sum\limits_{i,\:j}Var\left[{\xi\:}_{ij}\left|{A}_{ij}^{mf}\right|\right]\\&=2\:{\left(1\:W\right)}^{-2}\:{\Delta\:}x\:{\Delta\:}y\:{P}^{sample}\sum\limits_{i,\:j}E\left[{\left({\xi\:}_{ij}\left|{A}_{ij}^{mf}\right|\right)}^{2}\right]\\&=2\:{\left(1\:W\right)}^{-2}\:{\Delta\:}x\:{\Delta\:}y\:{P}^{sample}\sum\limits_{i,\:j}E\left[{\left({\xi\:}_{ij}\right)}^{2}\right]{\left|{A}_{ij}^{mf}\right|}^{2}\\&={\left(1\:W\right)}^{-2}\:{\Delta\:}x\:{\Delta\:}y\:{P}^{sample}\sum\limits_{i,\:j}{\left|{A}_{ij}^{mf}\right|}^{2}\\&={\left(1\:W\right)}^{-1}\:{P}^{sample}\end{aligned}$$

Here, we have used that the variance *Var* [*] of a sum of random variables *ξ*_*ij*_ with weights $\:\left|{A}_{ij}^{mf}\right|$ is equal to the sum of the variances of the weighted random variables (variance of sum rule), and that *Var* [*ξ*_*ij*_] = *E* [*ξ*_*ij*_^2^] – *E* [*ξ*_*ij*_]^2^ = *E* [*ξ*_*ij*_^2^] (since *E* [*ξ*_*ij*_] = 0). Finally, the expectation value of the modal power resulting from the noise seeding becomes *E* [*P*_*m*_] = 1 W × |*A*_*m*_|^2^ = *P*^*sample*^.

Insofar as the sample amplitudes *A*_*ij*_ and thus their complex conjugates *A*_*ij*_^*^ are normally distributed complex random variables with zero mean, the weighted sum is also a normally distributed complex random variable with zero mean. This includes the modal amplitude described by Eq. (8). Also note that since the complex distribution of *A*_*ij*_ is circularly symmetric, the phases of the weights $\:{A}_{ij}^{mf}$ (e.g., in case of a Gaussian beam with curved wavefront) do not matter.

From this, it follows that a sampled random field with one noise photon per sample will excite a mode with one noise photon (*n*_*sp*_ = 1). The results extend also to other values of *n*_*sp*_, and to any normalized distribution in the paraxial approximation, including Hermite-Gaussian modes and plane-wave modes, as well as non-modal distributions.
