# Supplementary material for: Modeling and simulation of amplified spontaneous emission in single-mode-pumped Cr2+:ZnSe bulk amplifiers with beam propagation and equivalent input-noise seeding
Source: Appl Phys B. 2025 Mar 1;131(3):63. doi: 10.1007/s00340-025-08433-y (PMC11870925; doi:10.1007/s00340-025-08433-y)
Supplement: Supplementary file 1 — Supplementary Material 1 [file 340_2025_8433_MOESM1_ESM.pdf]

Modeling and simulation of amplified spontaneous emission in single-mode-pumped Cr^{2+}:ZnSe bulk amplifiers with beam propagation and equivalent input-noise seeding: supplemental document

We here discuss the numerical grid, computational burden, n_{sp}, input-end and distributed noise seeding, and an alternative transfer matrix approach. We also discuss the calculation of the ASE spectrum, effective bandwidth, power, and self-saturation. We also evaluate the level of thermally generated light, which is found to be negligible, and discuss the effects of reflections and boundaries.

Numerical grid

The requirements on the numerical grid for BPM have been discussed in [1], [2], [3], and elsewhere. In our study, the grid must accommodate not only the propagation of the pump and signal beams, but also the ASE. The ASE is more difficult to predict, but it is possible to assess the adequacy of the grid by comparing it to the spatial and angular extent of the ASE after this has been calculated. See, e.g., Fig. 4. The spontaneous emission is different since it occurs in all directions, including those outside a paraxial description. However, the grid effectively truncates the spontaneous emission to angles it supports. The process is quantitatively straightforward, and it is straightforward to reproduce it experimentally. Thus, the spontaneous emission does not influence the choice of grid parameters. It is also possible to reduce the spatial frequency of the spontaneous emission and thus restrict its range of propagation angles in the simulations [3], but we did not explore this option.

Disregarding aberrations in the crystal, the pump and signal beams employed in the non-waveguiding crystals are diffraction-limited gaussian beams. Consider first the angular-spectrum (= spatial-frequency) domain. The half-width beam divergence parameter (at e^{-2} intensity) becomes $\theta_0 = \lambda / (n \pi w_0)$. In the paraxial approximation, inside the crystal, the maximum angle supported by the grid becomes $\theta_g = \lambda / (2 n \Delta x) = 0.492$ µm / Δx [rad] in the x-direction at the signal wavelength. Furthermore, $\theta_g / \theta_0 = \pi w_0 / (2 \Delta x)$. For Case A & B (confocal pumping), the smallest value of the waist radius used in the simulations was 23 µm. This was for the signal, whereby $\theta_0 = 13.6$ mrad. The sample spacing Δx (= Δy) of 16 µm that we used leads to $\theta_g = 30.7$ mrad. Comparisons to simulations with denser spacing and inspections of plotted angular spectra confirmed that the grid was adequate for the pump and signal.

In general, the ASE does not form a diffraction-limited Gaussian beam. However, under specific conditions, such as when the pump excites ions and generates significant gain beyond the pump focus at the crystal midpoint, Gaussian-beam calculations can still provide reasonable predictions for the grid requirements of the ASE. In this scenario, the ASE is dominated by "ASE rays" experiencing strong amplification and mostly confined to trajectories with comparatively high gain. The good beam quality observed with confocal pumping at pump powers above ~8 W supports this argument. The range of propagation angles of the ASE can then be approximated to that of the pump. By contrast, at low powers, the pump absorption length becomes only 1.93 mm in Case A. The input-face pump radius w becomes 0.0648 mm, so if the gain is high and dominated by the gain achieved in the first absorption length then this could result in ASE traveling at angles of up to 2 × 0.0648 / 1.93 rad = 67.2 mrad. These would not be supported by the grid. Yet, in Case A, it was not possible to reach gain high enough for ASE to dominate over the omni-directional spontaneous emission without having significant gain beyond the crystal midpoint.

In addition, however, at sufficiently high pump power, the pump intensity becomes high enough to create high gain and thus ASE beyond the e^{-2} intensity confines of the pump beam. This increases the range of angles over which the gain can be high. Ultimately, the adequacy

of the angular range for the ASE was verified through comparisons to simulations with denser sampling as well as through inspection of divergence plots like those in Fig. 4.

In Case B, the higher concentration made it possible to reach high gain already before the pump focus at the crystal midpoint, and the truncation imposed by the grid may have affected the results to a small degree for pump powers around 5 W. At this point, the agreement with Eq. (2) was poor, but simulations with an enhanced grid did not improve the agreement. Therefore, the lack of agreement was not caused by an inadequate grid in this case, either.

As it comes to the spatial domain, the ASE is largely contained within the pump caustic for similar “ASE ray” reasons. In turn, the pump was well contained within the grid window of 512 μm × 512 μm used for Case A & B.

With tight pump focusing (Case D, $w_{0,\mathrm{p}} = 8$ μm), we used $\Delta x = \Delta y = 8$ μm sample spacing. This may be marginal in the spatial-frequency domain, but inspection showed that the pump intensity drops by over three orders of magnitude from the center to the edge of the grid. The optimal focusing of the signal is less tight than that of the pump, so therefore better contained in the spatial-frequency domain. Also the ASE was reasonably well contained in the spatial-frequency domain. This is expected from the same “ASE-ray” argumentation as for Case A above. In the spatial domain, similar simple calculations suggest that the different waves are well-contained by the 2048 μm × 2048 μm window, and this was confirmed by inspection.

The large pump radius in Case C ($w_{0,p} = 200$ μm) is different and results in high-gain “ASE rays” at angles much larger than the angular spread of the pump beam. The angle of a ray from the edge (e^{-2} intensity-point) of a gaussian beam at the input facet to the diametrically opposite edge at the output facet is given by $w_0\,[(1 + (d_{in}/z_{R,c})^2)^{1/2} + (1 + (d_{out}/z_{R,c})^2)^{1/2}] / (d_{in} + d_{out})$. Here, d_{in} and d_{out} are the distances from the focus (assumed to be inside the crystal) to the input and output facets and $z_{R,c}$ is the Rayleigh length in the crystal. With midpoint pump focusing (i.e., $d_{in} = d_{out}$) the expression simplifies to $w_0\,(d_{in}^{-2} + z_{R,c}^{-2})^{1/2} = 26.6$ mrad for Case C. Angularly, this is well contained with the sample spacing of 8 μm that we used. All waves are also spatially well-contained by the 2048 μm × 2048 μm window.

The use of Fourier transformation for the diffractive part of the propagation step makes the structure effectively periodic in the two transverse dimensions. To prevent this artificial periodicity from influencing the propagation, it is common to use an absorber at the lateral boundaries. However, we did not do that since it would complicate the nearfield and farfield calculation and subtraction of the residual equivalent input noise. An alternative is to make the window sufficiently large so that at least, emission generated and potentially amplified in the pumped part of the gain medium, centrally in a window, does not leak into the artificial adjacent windows. It follows that the window width $N\,\Delta x$ should exceed $2\,L\,\theta_g = L\,\lambda / (n\,\Delta x) = 2.1$ mm with $\Delta x = 8$ μm (Case C & D). We consider that the 2048-μm grid satisfies this for Case C & D. In Case A & B, $\Delta x = 16$ μm so $2\,L\,\theta_g = L\,\lambda / (n\,\Delta x) = 1.0$ mm. The window of 512 μm × 512 μm did not fulfil this but was still large enough for the high-gain region to be effectively separated from high-gain regions in adjacent windows. Moreover, trials with a 1024 μm × 1024 μm window did not yield significant differences.

The longitudinal stepsize Δz must be small enough to ensure that the difference in phase change $\Delta\varphi$ between samples in a step is smaller than π. Normally, this is analyzed in terms of some refractive-index variation Δn of a waveguide structure [1], [2]. In our fiber simulations, this is quite small, $\Delta n = 2\times10^{-4}$, so this dictates $\Delta z < \pi / \Delta k = \lambda / (2\,\Delta n) = 4.8$ mm (for the pump). This was well fulfilled by our numerical grid.

Moreover, and even in the absence of a waveguide, there is a phase change relating to the diffractive part of a step, so the phase difference between rays (or plane waves) traveling at different angles (i.e., with different transverse wavenumbers) must be smaller than π. Although the maximum relevant propagation angle (e.g., of a Gaussian beam) is generally smaller than the maximum angle θ_g supported by the grid, we here use $2^{1/2}\,\theta_g$ for assessing Δz, to cover also spontaneous emission traveling diagonally at 45° in the x–y-plane. The (maximum) value of $\Delta\varphi$ becomes $\Delta z\,2\,\pi\,n\,(1 - \cos 2^{1/2}\,\theta_g) / \lambda \approx \Delta z\,2\,\pi\,n\,\theta_g^2 / \lambda = \Delta z\,\pi\,\lambda / (2\,n\,(\Delta x)^2)$. Thus, $\Delta z = \Delta\varphi$

$2\, n\, (\Delta x)^2 / (\pi\, \lambda) < 2\, n\, (\Delta x)^2 / \lambda = 260$ µm with $\Delta\varphi < \pi$ and $\Delta x = 8$ µm for Case C & D. The 100-µm stepsize of Case C & D fulfilled this. The other cases, as well as the fiber simulations used a stepsize that fulfilled $\Delta\varphi < \pi$ with larger margin.

In addition to the difference in phase change, the gain in a step must be sufficiently small, as well. Given the 1.35 dB/mm maximum gain and the 100-µm stepsize of Case C and D, this translates to 3.2% per step. A Taylor expansion of exponential growth suggests that this may underestimate the actual gain by 1.6% (0.139 dB/step instead of 0.135 dB/step), which we deem acceptable. The gain per step and thus the corresponding error was slightly smaller in case A and B. Moreover, the software may use corrections that further reduce the gain error, although this is not known to us. Trials with shorter stepsize did not lead to significantly different results.

Reflections at end-faces and lateral boundaries

The high refractive index of Cr^{2+}:ZnSe (~2.45) leads to high Fresnel reflections, ~17.7% at perpendicular incidence, and a critical angle for total internal reflection of 24.1°. Insofar as these affect the amplification and ASE, they should be included in the simulations. On the other hand, they are generally detrimental, so amplifiers are typically configured to suppress them.

End-face reflections can be particularly detrimental. Therefore, end-faces are typically anti-reflection-coated. However, even with a coating, residual reflections of signal and ASE would be problematic if they experience the full gain of the Cr^{2+}:ZnSe, which can exceed 40 dB in our simulations. To avoid this, the end-face can be angled. The suppression of amplification of reflected light is aided by the small angular extent of the pumped volume, thanks to single-mode pumping. For the reflected ASE to be negligible, the level should be small relative to the equivalent noise seeding of one photon per gridpoint (valid in spatial as well as spatial-frequency domain).

We discuss this in relation to case A with 100 W of confocal pumping. In this case, the signal gain becomes 41.8 dB and the total number of ASE-photons at the output becomes 43,360, equivalent to 46.37 dB and averaging to 42.3 photons/gridpoint. To simulate this, the ASE could be reflected with appropriate reflectivity. Simulations of bidirectional ASE is then required. This is beyond the scope of this paper, especially if there is ASE self-saturation. However, if the reflectivity is such that the number of photons that are reflected into the high-gain region (which generates the ASE) is small compared to the equivalent noise seeding then the reflection can be neglected. To assess this, Fig. S1 plots the ASE PSD in terms of photons per gridpoint in the spatial-frequency domain *vs.* the angle of propagation to the optical axis. The broadening of the curve is a consequence of different number of ASE-photons in different directions in the x-y plane (perpendicular to the optical axis). The ASE PSD reaches 799 photons in the central gridpoint, suggesting that a reflectivity of $\sim 10^{-4}$ would be required to make reflected ASE negligible. However, such low reflectivities are difficult to reach. It is then possible to angle the crystal, so that ASE parallel with the optical axis and the pump beam gets reflected at an angle, thus largely avoiding the high-gain region. For an approximate assessment, Fig. S1 shows that for an angle of 1°, the PSD is reduced to 10 photons/gridpoint. If the end-face is angled so that a 1° propagation angle is perpendicular to the end-face then a reflectivity of $\sim 10^{-2}$ is sufficiently low to make the reflected ASE at this angle much smaller than the noise-seeding.

Other spatial frequencies experience a change of propagation angle upon reflection in the angled end-face. It is particularly relevant to ensure that the high-intensity light parallel with the pump beam on incidence is reflected out from the pumped region. Most of this light overlaps with the high-gain region on incidence, and also immediately following reflection. Given a reflectivity of no more than 1%, this is unlikely to locally compress the gain to any significant extent. However, if the ASE overlaps with the high-gain region over a significant distance, it can still see significant gain and potentially deplete the pump and thus compress the gain. The diameter of the ASE was ~205 µm at the e^{-2} intensity point. The distance over which reflected light (no longer parallel with the incident pump beam) has significant overlap with the pumped

region can be estimated to 205 μm / (2 θ_{EF}), where θ_{EF} is the angle of the end-face. If θ_{EF} = 17.45 mrad (i.e., 1°) then the distance becomes 5.87 mm. This is over 30% of the crystal which may be excessive, but an angle of 0.1 rad (5.73°) reduces the distance to ~1 mm which seems small enough. This justifies ignoring this effect, but we also point out that the grid we use would not support the propagation angle of 0.2 rad that results following reflection with θ_{EF} = 0.1 rad.

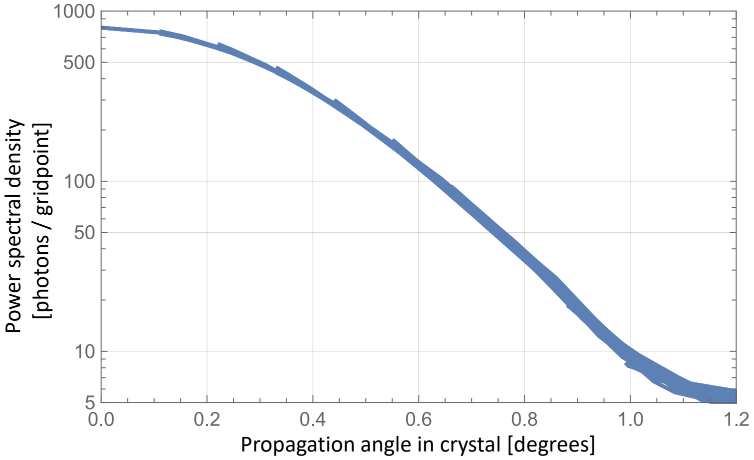

Fig. S1. ASE PSD at the output of the Cr^{2+}:ZnSe-crystal in spatial-frequency domain *vs*. propagation angle for Case A with 100 W of pump power.

Consider now the total number of reflected ASE photons, which could also be a problem if they seed backward ASE in a random fashion, potentially leading to self-saturation. There are 43,360 incident photons (S_{ASE} = 3.57 fW/Hz), and with 1% reflectivity we get 433.6 reflected ASE photons. This is smaller than the 32^2 = 1024 photons in the equivalent input noise, but not much smaller. To remedy this, it is possible to increase the number of gridpoints by making the computational window larger, say, doubling it from 512 μm to 1024 μm, whilst maintaining the sample spacing of 16 μm. The number of ASE-photons on output should not change (following REIN subtraction), since already the original window was large enough to accurately calculate S_{ASE}. However, there are now more than 4096 photons in the equivalent input noise, which is much more than the reflected ASE-photons. Since the cross-sectional area of Cr^{2+}:ZnSe crystals is typically larger than 1024 μm × 1024 μm, that this category of reflected ASE is not a cause of concern for Case A.

Reflections at lateral boundaries could also be a problem. However, the computational windows of 512 μm and 2048 μm were chosen to ensure that strong ASE would not be reflected back into the pumped region, and the cross-sections of real crystals are unlikely to be smaller than 2 mm × 2 mm. Furthermore, single-mode pumping reduces the pump volume e.g., to 0.075 mm^3 in Case A. This is three orders of magnitude smaller than the 68 mm^3 volume of a 2 mm × 2 mm × 17 mm crystal. Altogether, the small pump volume enabled by single-mode pumping makes it possible to suppress and hence ignore the effects of boundaries in a properly configured 17-mm-long Cr^{2+}:ZnSe crystal with 42-dB of gain (Case A).

With higher gain, e.g., as was achieved in the simulations of Case B, the effects of boundaries can be significant (especially end-face reflections). We also reiterate that self-saturation occurred in Case B at gain > 50 dB. We have used grids with transverse window up to 2048 μm, so actual crystals with such transverse dimensions can be simulated. This requires an appropriate description of the lateral boundary. If this is polished, light within the angular range of the computational grid will undergo total internal reflection. This is straightforward to simulate, and the small pumped volume suggests that the effect of such light will be negligible. Indeed, this was found to be the case in Case B with its 512-μm window, where the periodic

boundary conditions of Fourier-transform-based BPM lead to similar requirements on the window size as possible reflections do.

Alternatively, an absorbing boundary is straightforward to implement in BPM, although in case of ASE-simulations, the impact on the REIN needs to be considered.

Reflecting as well as absorbing lateral boundaries are straightforward to implement in RP Fiber Power [4], provided that the boundaries lie in planes with constant x or y. Optionally, the propagation may be at an angle to the z-axis.

We do not consider a scattering boundary, but note that small pumped volume with single-mode pumping means that scattered light sees much lower levels of gain than beams within the gain region.

There is also a question if spontaneous emission emitted at large angle to the direction of propagation can propagate in a zig-zag pattern and accrue significant gain as it does so. This would not be included in our simulation, if the propagation angle (or transverse wavenumber) exceeds that supported by the grid. The simple answer is no, since the small pumped volume makes the average overlap with the gain region, and thus the gain, of any zig-zagging lightwave small.

In summary, we emphasize that the small gain volume with single-mode pumping greatly reduces the problems related to boundaries, in the simulations as well as in reality. Nevertheless, if sufficiently high gain can be reached, the boundaries will become important in simulations as well as in reality.

n_{sp}

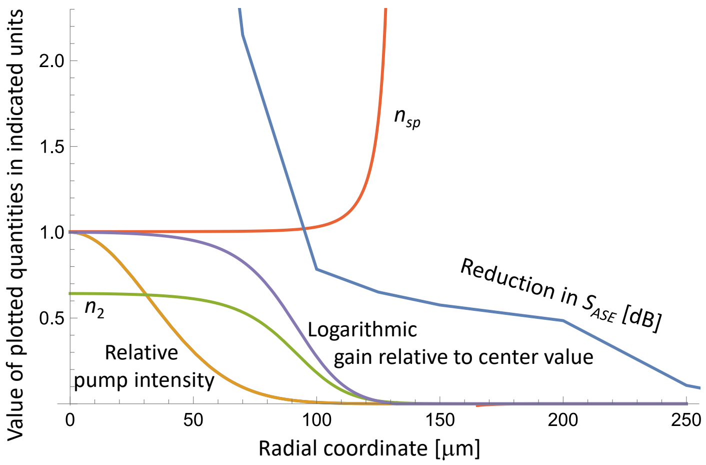

Fig. S2. Relative pump intensity, relative logarithmic gain, n_{sp} according to Eq. (3), and n_2 *vs*. radial coordinate in the crystal input plane for Case A with 50 W of pump power. Also plotted is the reduction in S_{ASE} that results if the input noise seeding is restricted to a circular disk within the radial coordinate instead of n_{sp} = 1 everywhere.

Our BPM simulations use one noise photon per gridpoint, corresponding to n_{sp} = 1, but any signal background loss or ground-state absorption makes it larger. See Eq. (3). Note also that Eq. (3) is simplified, since n_{sp} depends on the longitudinal distribution of n_2 (e.g., [5]). On the other hand, n_{sp} is straightforward to evaluate analytically in some cases. In our fiber simulations, a pump power much higher than the saturation power throughout the doped region and the lack of background loss means that n_2 reaches its pump transparency level of $n_{2,PT}$ = 0.657, and that Eq. (3) applies with $n_{sp} = \sigma_d^p \sigma_e^s / (\sigma_d^p \sigma_e^s - \sigma_d^p \sigma_e^s)$ = 1.0035. The difference to our assumption of n_{sp} = 1 is only 0.015 dB. However, in the bulk amplifiers with limited pump power, these conditions do not apply. The fractional excitation n_2 may be below the pump transparency level everywhere, especially in regions with low pump intensity. Thus, n_2 varies longitudinally as

well as between samples within the input grid. This makes the calculation of n_{sp} more complicated. Nevertheless, Fig. S2 shows n_{sp} calculated according to the simple Eq. (3) and n_2 *vs*. radial coordinate r at the input plane for Case A with 50 W of pump power. The input gain [dB/m] and the pump intensity are also shown, scaled by their values at $r = 0$. The pump radius w is 64.8 μm at the input plane. In addition, Fig. S2 shows the reduction in S_{ASE} if the ASE is only seeded at radii smaller than the radial coordinate in question, i.e., if n_{sp} is changed from one to zero for larger radii. For example, compared to our usual BPM simulation approach with the ASE seeding extending over the full input grid (512 μm × 512 μm in this case), S_{ASE} drops by 0.7 dB if the ASE-seeding is restricted to the circular region $r < 110$ μm. In this region, $n_{sp} < 1.1$ according to Eq. (3), making $n_{sp} = 1$ a reasonable approximation. Outside 110 μm, Eq. (3) leads to a rapidly increasing value of n_{sp} but given the relative insensitivity of S_{ASE} on the ASE-seeding outside 110 μm in this example, this is not expected to be significant for S_{ASE}.

Fig. S2 also shows that n_{sp} diverges at a gain of 0 dB (corresponding to $G_{lin} = 1$). However, since $(G_{lin} - 1) \rightarrow 0$, Eq. (1) does not diverge. Instead, $S_{ASE} / (h\nu) \rightarrow L\ N_0\ n_{2,ST} = 0.1036$ photons/gridpoint for the length L and dopant concentration N_0 of the crystal in Case A, and with the crystal uniformly excited to the signal transparency level $N_0\ n_{2,ST} = \sigma_a^s / (\sigma_a^s + \sigma_e^s) = 0.00670$. This agrees within 10% with a BPM simulation under these conditions and with $n_{sp} = 1$. The BPM simulations resulted in $S_{ASE} / (h\nu) = 95.1$ photons in 32×32 gridpoints, thus 0.0929 photons/gridpoint (following REIN subtraction). From this, we conclude that even a significant amount of weakly pumped gain medium would not change n_{sp} that much (e.g., by 10%), which further supports the conclusion that the error in S_{ASE} induced by the approximation $n_{sp} = 1$ also in weakly pumped regions is small.

Effective bandwidth of ASE and evaluation of ASE self-saturation

To assess self-saturation (by ASE) in our bulk-crystal BPM simulations, we crudely simulated the total ASE power in the forward direction P_{ASE} in the same way as we did S_{ASE} (= power in 1 Hz) at 2410 nm, i.e., as a monochromatic wave with ensemble-averaging of the power and REIN subtraction, but with the equivalent-input noise seeding scaled by the effective ASE bandwidth $\Delta\nu_{eff}$ and the two polarizations. At the same time, we calculated the gain of a weak signal. The ASE self-saturation may change the optimum signal focusing, but this was not considered.

The effective bandwidth depends on the gain and was calculated from a spectral integral of Eq. (1), i.e., the total ASE power in a single spatial mode:

$$P_{ASE} = 2 \int_{\substack{ASE \\ spectrum}} S_{ASE}(\nu)\, d\nu = 2 \int_{\substack{ASE \\ spectrum}} (G_{lin}(\nu) - 1)\, h\nu\, n_{sp}(\nu)\, d\nu = 2\Delta\nu_{eff}\left[(G_{lin} - 1)\, h\nu\, n_{sp}\right]_{2410\,nm} = 2\Delta\nu_{eff} S_{ASE,2410\,nm} \quad \text{Eq. (S1)}$$

Here, the values of G_{lin} and n_{sp} depend on the optical frequency ν as well as on the fraction of excited Cr^{2+}-ions n_2, which was fixed to yield a specific gain at 2410 nm in the crystal under consideration. In the integrand, G_{lin} and n_{sp} were then calculated for that value of n_2. Then, $\Delta\nu_{eff} = P_{ASE} / \left(2\, S_{ASE,2410\,nm}\right)$. Fig. S3 plots P_{ASE} evaluated with Eq. (S1) *vs*. S_{ASE} at 2410 nm evaluated with Eq. (1). The relation is close to a line according to $P_{ASE} \approx (S_{ASE} / h\nu) \times 2.5$ μW/photon for gain up to 50 dB for the crystal of Case A, C, D (and the cross-section spectra of Cr^{2+}:ZnSe). This corresponds to an effective linewidth of 15 THz for this crystal. More precisely, the effective linewidth becomes 35.6 THz (690 nm) for $G_{lin} \rightarrow 1^+$, 14.6 THz (282 nm) at a gain of 44.0 dB (the highest that can be reached with 1901-nm pumping), and 11.6 THz (224 nm) at the fully inverted gain of 67 dB ($n_2 = 1$). Further calculation details are as follows. Generally, G_{lin} depends on the value of n_2 (as averaged for a specific mode or signal light distribution) in a one-to-one relation for specific crystal parameters, namely, G_{lin} = exp

$\{N_0\, L\, [(\sigma_e^s + \sigma_a^s)\, n_2 - \sigma_a^s)]\}$. Furthermore, the gain is equal in the forward and backward direction. By contrast, n_{sp} depends on the distribution of n_2. As a consequence, the ASE in the forward and backward direction are typically different. With input-end (or rather external) noise-seeding of the ASE, the backward ASE is seeded from the output end (for both pump and signal in our case). Since n_2 is larger in the input end with co-directional signal and pump propagation, this means that n_{sp} is larger for the reverse (backward) direction and thus that the backward ASE is stronger than the forward ASE. Nevertheless, for simplicity, in Eq. (S1) we use the average value of n_2 also for calculating n_{sp}, according to Eq. (3). This also relates any two of the quantities n_2, $G_{lin}(\nu)$, $n_{sp}(\nu)$, $S_{ASE}(\nu)$, P_{ASE}, and $\Delta\nu_{eff}$ to each other, for a specific set of crystal parameters, and provided that the difference in propagation (e.g., rate of diffraction) at different ASE-wavelengths can be neglected.

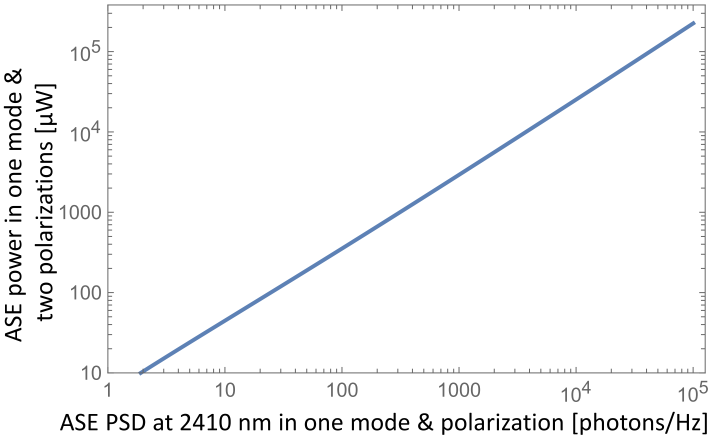

Fig. S3. ASE power in one mode and two polarizations according to the second integral in Eq. (S1) for the crystal of Case A, C, D *vs.* S_{ASE} in one mode and one polarization at 2410 nm according to Eq. (1).

We ran a selection of the BPM simulations in Sect. 4 with the noise seeding scaled by an effective bandwidth determined as described here, and by the two polarizations, to assess the self-saturation. The fact that ASE affects the population inversion in the self-saturation regime suggests that the residual equivalent input noise should be subtracted whenever the inversion is calculated. It is easy to show that n_{sp} photons per polarization per second per hertz over the full emission bandwidth leads to a net relaxation rate which is equal to the spontaneous-emission rate multiplied by the fractional solid angle that the numerical grid represents (forward and backward direction), so a fraction of the spontaneous emission is potentially counted twice in the rate equations. Our BPM grid corresponds to only a small fraction of the total solid angle, given by $(\lambda / (n^2\, \Delta x\, \Delta y)) / (4\,\pi) = 1.2\times10^{-3}$ in one direction for $\Delta x = \Delta y = 8$ µm. It follows that the transition rate corresponding to the REIN is negligible compared to the spontaneous-emission rate. Therefore, the error resulting from the inclusion of the REIN in the rate equations when calculating the ASE self-saturation is negligible.

The ASE self-saturation led to a compression of the signal gain at 2410 nm of < 0.05 dB for all pump powers in Case A, C, D. This was with forward-propagating ASE, but even if this may more than double with bidirectional ASE, it can still be neglected. However, with the crystal in Case B, self-saturation becomes significant at ~50 dB of gain. (This gain level was never reached in the other cases with 1901-nm pumping.) Note also that regardless of any simulation issues, it is still possible to use measured (as well as accurately simulated) values of M_{ASE}^2 and S_{ASE} to calculate the gain according to Eq. (2), which may remain valid in the self-saturated regime (depending on factors such as the shape of the saturated gain distribution).

We also point out that in the self-saturating regime, n_{sp} and the ASE-power depend on each other and need to be determined self-consistently.

Outline for the evaluation of bi-directional ASE spectra with self-saturation

We did not calculate any ASE spectra but will briefly comment on options for this. The ASE spectrum can then be integrated to calculate the ASE power more accurately than with the effective-bandwidth approach that we used in our estimate of the ASE self-saturation. To calculate the spectrum and its spatial propagation, the ASE can be divided into a number of "spectral bins", as it commonly is for MPE-simulations of fiber amplifiers. See, e.g., [4], [5], [8]. With BPM, both distributed and input-end noise seeding can be used. In each bin, the ASE is represented by a monochromatic wave. Insofar as the ASE is strong enough to saturate the gain, we must simultaneously simulate enough bins to accurately represent the saturation. Typically, ASE in both directions contributes to the saturation and must then also be simulated simultaneously (with incoherent contra-directional bins even if they are at the same optical frequency). Each ASE-bin is seeded with noise of random phase and amplitude that makes the expectation value of the seed power proportional to the spectral width (which may differ between bins) in each gridpoint, either at the ends of the crystal or in a distributed manner. We reiterate that the noise seeding (i.e., n_{sp}) for ASE counter-directional to the pump is typically larger than that for co-directional ASE. Since the field of an ASE bin is monochromatic, it will form a random interference pattern with hotspots like the pattern shown in Fig. 4 (b). If the power in a single bin is excessive, its interference pattern will result in a similar spatial pattern in the gain saturation, which will enhance the saturation for that ASE-bin. Experimentally, however, the instantaneous interference pattern of the ASE varies sufficiently fast in time for the pattern to be averaged out over time-scales relevant for the population inversion. This is very roughly of the order of 1 μs for Cr^{2+}:ZnSe. We expect that if the power of a single bin carries no more than ~1% of the total ASE power in simulations then such artificial interference effects will be small due to averaging of the patterns caused by different spectral bins. This suggests that 100 bins can be enough, and possibly significantly fewer, with an appropriate selection of bin parameters. As before, the ASE spectral power density S_{ASE} can then be evaluated as an ensemble-average, separately for each bin. In case of input-end seeding, the residual equivalent input noise needs to be subtracted.

RP Fiber Power does support propagation of multiple waves ("optical channels"), each of which can correspond to an ASE-bin [4]. However, depending on the details of the implementation, memory or other restrictions may prevent the use of 100 ASE-bins. Also the computational burden of BPM simulations can easily become prohibitive, and ASE saturation can slow down convergence or even thwart it. For a single pass through the crystal, the BPM run-time scales approximately with the number of ASE bins. In our simulations with a single forward ASE wave, a single realization took between 0.5 and 5 s to run on a workstation, depending on transverse and longitudinal grid size. Some of this is overhead, but scaling in proportion to the number of waves suggests runtimes of between 25 and 250 s in case of saturating ASE. However, more passes may well be required if the rate of convergence is reduced. Averaging over 100 runs might take approximately 1 – 10 hours.

There are ways to reduce the computational burden, with bespoke code and perhaps also with commercial software with advanced scripting such as RP Fiber Power. The requirement that the instantaneous interference pattern of a bin does not affect the saturation suggests that the spatial gain distribution will not vary between realizations, for a sufficiently large number of bins. It is then possible to calculate the gain distribution once and for all and use that gain distribution to propagate a low-power ASE wave. This can then be ensemble-averaged over several runs to obtain the spatial distribution and level of the ASE at one or several wavelengths in the saturated regime. In case of sparse spectral sampling, interpolation can be used to estimate the ASE at intermediate wavelengths. Generally, conventional interpolation cannot be viewed as a way to improve spectral resolution. However, if the absorption and emission cross-

section spectra are available with high resolution then it may be possible to use those to improve the accuracy of interpolated values, and thus arguably improve the spectral resolution of the ASE. Conversely, if the bin density exceeds the required spectral resolution, it is possible to group, e.g., five bins together and that way reduce the number of runs required for the ensemble-averaging.

The memory required depends on the details of the implementation. A bare minimum is a complex number per transverse gridpoint and wave in the propagating waves plus the same number of complex numbers in each end of the crystal to store the output waves of the previous iteration (needed to assess convergence). With 256×256 transverse points, 100 waves, and 16 bytes per complex number, we get $3\times256^2\times100\times16 = 315$ MB. In addition, the total excitation and relaxation rates induced by the waves propagating in the opposite direction must be stored in each transverse and longitudinal gridpoint. With 200 steps and 8 bytes per real number, this becomes $256^2\times200\times2\times8 = 210$ MB. These are modest requirements and implementations that store all waves in all transverse and longitudinal gridpoints may also be realistic (e.g., $256^2\times200\times100\times16 = 21$ GB).

A run with a single ASE, pump, and signal wave and an estimated minimum storage of $6\times256^2\times16 + 256^2\times200\times2\times8 = 216$ MB actually required 1060 MB in RP Fiber Power. However, a large fraction of this is overhead and we have not assessed how the memory usage scales with the number of ASE waves.

If ASE self-saturation is negligible then any bin-count restriction can be overcome by simulating bins sequentially. Interpolation enhanced by the cross-section spectra may then offer a route to increased spectral resolution with a relatively small number of bins. In any case, for the simulation of an ASE spectrum with or without saturation, we expect that runtimes will be more restrictive than memory requirements.

Counter-propagating waves which both affect the population inversion are nonlinearly and nonlocally coupled by the gain and / or thermal effects. This includes saturating bidirectional ASE, and can stop iterative algorithms from converging to steady-state solutions (e.g., if all solutions are unstable). In such cases, one can resort to solving time-dependent equations for the heating, thermal transport, nonlinearities, inversion, and wave propagation (including ASE). For this, it would be important to run the simulations long enough with the required temporal resolution to display relevant dynamics of the system with its different time constants, and to allow averaged quantities to be evaluated. The (average) properties of the ASE (e.g., spectrum, power, beam quality) can in principle be evaluated as a spectral, temporal, or ensemble average. The availability of sufficiently rapidly varying time-dependent ASE should make it possible to reduce the number of spectral ASE-bins needed in the simulations. Although conceptually relatively simple and not relying on convergence, this considerably more demanding case is beyond our scope.

Input-end *vs*. distributed noise seeding

Our results and estimates in Fig. S2 and elsewhere support the validity of the approximation $n_{sp} = 1$ in our BPM-simulations. However, with significant background loss or ground-state absorption of signal photons (e.g., in a three-level system), it is no longer a reasonable assumption. With co-propagating pump and signal it may be straightforward to calculate the spatially resolved fractional inversion and then approximate n_{sp} by Eq. (3) across the input plane (similar to Fig. S2). However, the validity would have to be established in this case. To avoid these issues, it may be better to distributively seed the ASE with spontaneous emission throughout the gain medium [3], [6], [7] (as for the MPE-approach). This also avoids the need to subtract the residual equivalent input noise. Although quite accurate in our simulations, the REIN subtraction may be less accurate with large and spatially varying n_{sp}.

The computational burden of a single run is largely the same with input-plane and distributed seeding of the ASE, since the burden is expected to be dominated by the Fourier transforms of the BPM propagation. However, the number of runs required for the ensemble-

averaging may differ. The use of equivalent input noise with subsequent subtraction of the REIN may require a larger number of runs for accurate ensemble-averaging, since the random noise seeding is concentrated to a smaller number of gridpoints and the REIN subtraction increases the relative size of the random fluctuations that need to be averaged out in the ensemble-averaging. The effect of this is particularly noticeable in regions where the REIN is a significant part of the output field. Thus, we attribute the large relative fluctuations away from the core in Fig. 3 and 4 to this. We have not tried to evaluate how it affects the required number of runs, but note that distributed noise seeding does not suffer from this. We reiterate that seeding with equivalent-input noise does not require the core of the BPM code to be modified.

Transfer matrix approach

A potential advantage with the equivalent-input noise approach is that in the absence of gain saturation, the BPM propagation and thus the complex amplitude becomes linear from input to output. In cases where the transverse grid is small, e.g., 32×32 = 1024 points, where $N = 32$ is the number of transverse points in one spatial dimension, it would be possible to calculate an overall transfer matrix connecting the complex amplitude of each input point to that of each output point, for a particular set of pump and crystal parameters. Note that the transfer matrix comprises $N^2 \times N^2$ elements so is quite large already with $N = 32$. Alternatively, it can be viewed as a 4th-order tensor with 32^4 elements t_{kl}^{ij}. The output is given by $A_{kl}^{output} = t_{kl}^{ij} A_{ij}^{input}$, where A_{ij}^{input} is the complex amplitude in the input plane and where terms with indices occurring twice are summed. In case of equivalent-input noise, A_{ij}^{input} becomes a normally-distributed complex random variable with zero mean, with well-known properties. For example, n_{sp} (or n_{ij}^{sp}) becomes proportional to the expectation value of $\left|A_{ij}^{input}\right|^2$ (which is equal to the variance of A_{ij}^{input}). Thus, $n_{ij}^{sp} = E\left[\left|A_{ij}^{input}\right|^2\right] = Var\left[A_{ij}^{input}\right]$. Furthermore, the expectation value for the number of output noise photons n_{kl}^{output} (i.e., ASE) becomes $n_{kl}^{output} = E\left[\left|A_{kl}^{output}\right|^2\right] = E\left[\left|t_{kl}^{ij} A_{ij}^{input}\right|^2\right] = \left|t_{kl}^{ij}\right|^2 n_{ij}^{sp}$. We can also calculate other statistical quantities of the noise such as higher-order moments.

The transfer matrix requires N^2 BPM simulation runs to construct, each with a single point excited in the input grid. (Other orthogonal sets would work, too.) For comparison, the alternative approach with averaging of the noise that we actually used requires less computation if the number of realizations (runs) for the average is smaller than N^2, which it was in most cases already with $N = 32$, and in all cases with larger N. However, even if the averaging approach we used is faster in most cases, it lacks the analytic quality of the transfer-matrix approach.

The transfer matrix can also be used with deterministic input, e.g., to calculate the gain of a non-saturating signal as well as for the optimization of the signal launch used to find the value of G_{lin} in Eq. (2). Disregarding the cost of setting up the transfer matrix (if it is already known), that approach requires a single matrix multiplication and will be more efficient than the BPM propagation if the number of steps N_z exceeds $N^2 / (2 \log_2 N)$. For $N = 32$ this becomes 102.4, which is approximately half of the number of steps we used for the bulk crystal. For $N = 256$ it becomes 4096, which is much larger than the number of steps we used.

The tentative attractions of a transfer matrix need to be weighed against the multiplication count. Furthermore, distributed ASE-seeding and nonlinear effects including ASE self-saturation cannot be handled by a transfer matrix from input to output.

Thermal generation of radiation is negligible

Thermal generation of radiation can occur directly (as in blackbody radiation) as well as through thermal excitation of the upper laser level followed by spontaneous emission. This is

not necessarily an issue when we compare different calculations of emission which all neglect such thermal generation, but it could matter for comparisons to experiments.

The wavelength of 2410 nm that we consider corresponds to a temperature $T = h\, c\, /\, (\lambda\, k_B)$ = 5970 K. This is much higher than the actual temperature of the pumped gain medium, which we estimate to less than 500 K. At 500 K, the relative thermal occupation (Boltzmann factor) of the upper laser level becomes 6.52×10^{-6}. (Here, the energy difference between the upper and lower laser level has been approximated by the signal photon energy. The signal is red-shifted relative to the energy difference, so the actual energy difference is larger.) The thermal occupation is negligible compared to that resulting from the pumping.

Also other parts of the Cr^{2+}:ZnSe crystal can generate thermal emission. If we take 350 K as a high estimate of the temperature of unpumped parts of the crystal then the relative thermal occupation of the upper laser level becomes 3.91×10^{-8}. Even though the pumped volume may be no more than 0.01% of the total Cr^{2+}:ZnSe-volume, it is clear that the thermal emission from unpumped parts of the crystal is negligible. Scattered light seems likely to lead to stronger radiation from nominally unpumped parts of the crystal.

To assess the thermal background, we model it as blackbody radiation at a slightly elevated room temperature of 300 K. The Boltzmann factor becomes 2.28×10^{-9}, and the PSD at 2410 nm in becomes 3.23×10^{-17} W Hz^{-1} m^{-2} sr^{-1} in one polarization. This is, for example, much smaller than the PSD shown in Fig. 3, even if integrated over a solid angle of $2\,\pi$ sr. In terms of photons, the blackbody PSD becomes 392 photons s^{-1} Hz^{-1} m^{-2} sr^{-1} in one polarization Compared to the PSD-levels of a few photons shown in Fig. 8 and similar, this is not necessarily negligible if detected with an area of 1 cm^2 and a solid angle of 5 sr, for example. However, such background is routinely subtracted.

References

[1] M. D. Feit and J. A. Fleck, "Light propagation in graded-index optical fibers," Appl. Opt. **17**, 3990-3998 (1978)

[2] J. Van Roey, J. van der Donk, and P. E. Lagasse, "Beam-propagation method: analysis and assessment," J. Opt. Soc. Am. **71**, 803-810 (1981)

[3] James W. Greene, “Paraxial wave-optics simulation of x-ray lasers”, Phys. Rev. E **48**, 3130-3160 (1993)

[4] RP Fiber Power, www.rp-photonics.com/rp_fiber_power.html

[5] E. Desurvire, *Erbium doped fiber amplifiers: principles and applications* (Wiley-Interscience, 1994)

[6] M. D. Feit and J. A. Fleck, "Wave-optics description of laboratory soft-x-ray lasers," J. Opt. Soc. Am. B **7**, 2048-2060 (1990)

[7] J. C. Garrison, B. Ritchie, H. Nathel, C. K. Hong, and L. Minner, “Wave-optics description of amplified spontaneous emission”, Phys. Rev. A **43**, 4941 (1991)

[8] E. Desurvire and J. R. Simpson, “Amplification of spontaneous emission in erbium-doped single-mode fibers”, J. Lightwave Technol. **7**, 835-845 (1989)
